# Systems Biology Approach Pinpoints Minimum Requirements for Auxin Distribution during Fruit Opening

**DOI:** 10.1016/j.molp.2019.05.003

**Published:** 2019-06-03

**Authors:** Xin-Ran Li, Renske M.A. Vroomans, Samantha Fox, Verônica A. Grieneisen, Lars Østergaard, Athanasius F.M. Marée

**Affiliations:** 1 Crop Genetics, John Innes Centre, Norwich NR4 7UH, UK; 2 Computational and Systems Biology, John Innes Centre, Norwich NR4 7UH, UK; 3 Centre of Excellence in Computational and Experimental Developmental Biology, Institute of Biotechnology, University of Helsinki, 00014 Helsinki, Finland; 4 Cell and Developmental Biology, John Innes Centre, Norwich NR4 7UH, UK; 5 School of Biosciences, Cardiff University, Cardiff CF10 3AX, Wales, UK

**Keywords:** auxin, mathematical modeling, polar auxin transport, fruit development, systems biology of patterning

## Abstract

The phytohormone auxin is implied in steering various developmental decisions during plant morphogenesis in a concentration-dependent manner. Auxin maxima have been shown to maintain meristematic activity, for example, of the root apical meristem, and position new sites of outgrowth, such as during lateral root initiation and phyllotaxis. More recently, it has been demonstrated that sites of auxin minima also provide positional information. In the developing *Arabidopsis* fruit, auxin minima are required for correct differentiation of the valve margin. It remains unclear, however, how this auxin minimum is generated and maintained. Here, we employ a systems biology approach to model auxin transport based on experimental observations. This allows us to determine the minimal requirements for its establishment. Our simulations reveal that two alternative processes—which we coin “flux-barrier” and “flux-passage”—are both able to generate an auxin minimum, but under different parameter settings. Both models are in principle able to yield similar auxin profiles but present qualitatively distinct patterns of auxin flux. The models were tested by tissue-specific inducible ablation, revealing that the auxin minimum in the fruit is most likely generated by a flux-passage process. Model predictions were further supported through 3D PIN localization imaging and implementing experimentally observed transporter localization. Through such an experimental–modeling cycle, we predict how the auxin minimum gradually matures during fruit development to ensure timely fruit opening and seed dispersal.

## Teaser

We investigate the mechanism leading to a spatially confined auxin minimum required for *Arabidopsis* fruit opening and seed dispersal. Our experiment–modeling cycle reveals a counterintuitive mechanism of its formation: efflux activity creates an auxin minimum strip that is accompanied by high auxin fluxes crossing it perpendicularly. This dynamical process can be associated with the maturation of the fruit.

## Introduction

Patterning through morphogens is considered one of the first triggers for correct tissue differentiation (Raspopovic et al., 2014, Wolpert, 2016). Cell differentiation hinges on the concept of genetic control, first elucidated for single cells by the pioneering work of Jacques Monod, an early advocate of a systems view of living cells (Ullmann, 2011). How the patterning of cell differentiation is controlled within a coordinated multicellular structure, however, leads us to go beyond “anything found to be true of *E. coli* must also be true of elephants, only more so” (Jacob and Philip, 1995). Alike elephants, plants are multicellular organisms, but with a development that keeps continuously unfolding, never losing its capability to plastically alter in response to environmental cues. It is therefore insufficient to characterize the cells in isolation, mathematical modeling being required to study the entire tissue and explore its emerging properties and functionality (Grieneisen et al., 2012). In plants, tissue fates and their progressive differentiation are steered by phytohormones and their downstream genetic targets. Distribution of the phytohormone auxin is facilitated by specialized proteins, such as PIN efflux transporters and influx transporters of the AUX1/LAX family (Swarup and Péret, 2012, Adamowski and Friml, 2015), with many additional transporters and processes capable of affecting auxin flows (Park et al., 2017). As a consequence of its rapid, and often polar, transport through plant tissues, auxin distribution can be quickly and drastically altered by modifications of the expression levels or cellular localization of these transport proteins (Grieneisen et al., 2012). In *Arabidopsis thaliana*, auxin has been implied in establishing and maintaining the root apical meristem through an auxin maximum at the stem cell niche (Sabatini et al., 1999), dynamically formed by means of an auxin reflux loop (Grieneisen et al., 2007). Dynamic auxin distribution is also involved in the phyllotactic patterning of the shoot apical meristem, where lateral organs emerge through auxin maxima that form as a consequence of neighboring PINs orienting toward these sites (Barbier de Reuille et al., 2006, Smith et al., 2006). Finally, auxin accumulation in root pericycle cells can trigger lateral root initiation sites (Benková et al., 2003, Dubrovsky et al., 2008), further amplified by the AUX1/LAX family (Marchant et al., 2002, Laskowski et al., 2008). In addition to its role in the positioning and initiation of whole organs (Reinhardt et al., 2003), localized auxin maxima are also crucial for the correct spacing of serrations at the edge of leaves (Scarpella et al., 2006, Bilsborough et al., 2011), root hairs (Payne and Grierson, 2009), and xylem-phloem poles (el Showk et al., 2015).

Given the wide implications of auxin maxima to plant development, the existence of auxin minima has been largely eclipsed or simply regarded as inevitable concentration valleys intercalating maxima. However, functional significance of auxin minima and their modes of regulation that can be independent from maxima have been emerging within several contexts, ranging from phyllotaxis to root development. Extended regions of low auxin have been shown to be instructive for maintaining crevices between meristems in the SAM and between leaf indentations (Stoma et al., 2008, Heisler et al., 2010, Caggiano et al., 2017). Furthermore, the regulated formation and maintenance of an auxin minimum at the basal root meristem triggers cell differentiation (Di Mambro et al., 2017). The first evidence of a functional auxin minimum stems from *Arabidopsis* fruits, where depletion of auxin from narrow strips of cells is required for seed dispersal (Sorefan et al., 2009). In contrast to localized auxin maxima, the mechanistic basis of how such a distinct minimum can be established is less clear (Grieneisen et al., 2013) and has not been confirmed experimentally.

**Figure 1:**
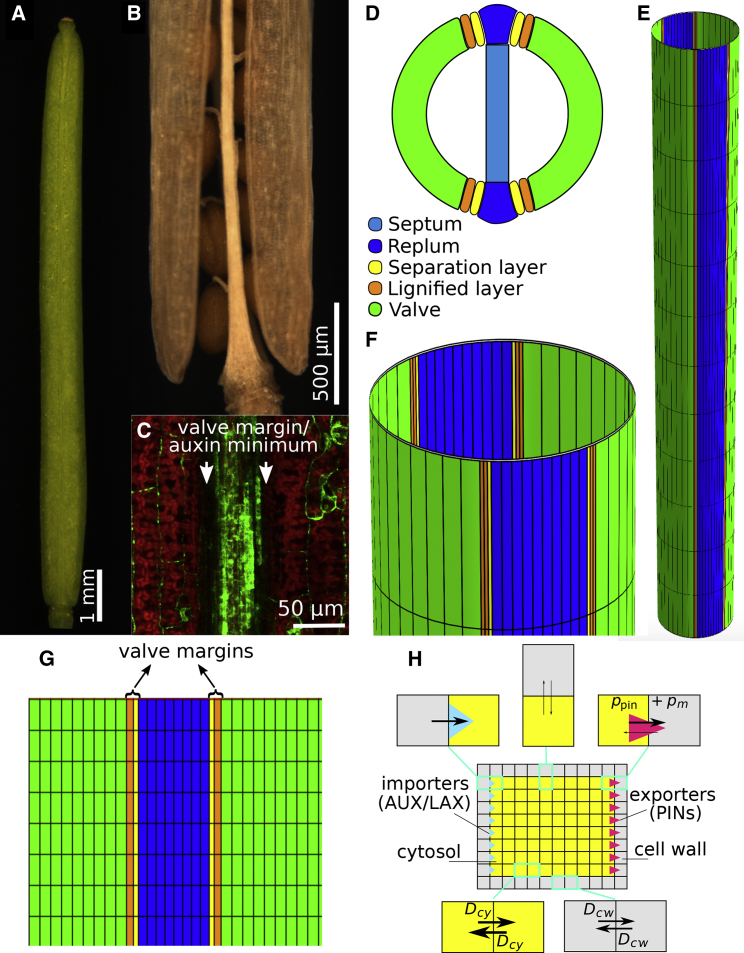
Modeling Auxin Transport in the Developing *Arabidopsis* Fruit. **(A)** Silique at stage 17b. **(B)** Dehiscence along the valve margin (VM) (stage 19). **(C)** Auxin-signaling minimum at the VM, shown by DR5:GFP expression. **(D)** Schematic transversal cross-section of the bilaterally symmetric ovary, with tissues indicated, also showing the internal septum that we do not simulate within this modeling framework. **(E)** Schematic of the cylindrical model layout of the external fruit tissues, visualizing the topological connectedness. **(F)** Zoomed-in portion of **(E)**, displaying approximately one cell row. **(G)** Schematic of the model layout of the longitudinal fruit, laid out in 2D, indicating all modeled tissue types through color coding. Note that here only half of the fruit tissue is displayed, whereas simulations were always done on the full, cylindrically connected tissue. **(H)** Within the model, auxin transport across plasma membrane as well as diffusion in cytosol and apoplast (cell wall) at subcellular resolution are taken into account.

*Arabidopsis* fruits develop into cylindrical siliques composed of two valves (seed pod walls) that are connected to a central replum (Figure 1A, 1B, and 1D). Internally, the replum is linked to the septum from which the seeds will develop (Figure 1D, light blue). Specialized cell types differentiate at the border between the valves and the replum, called valve margin (VM) cells (Figure 1D and 1G). Late in development, the VM tissue differentiates into dehiscence zones where cells eventually undergo cell death, allowing the valves to separate from the replum and release the seeds in a process known as fruit dehiscence (Figure 1A and 1B) (Roeder and Yanofsky, 2006). Prior to formation of the dehiscence zone, the VM cells undergo a cell division event that leads to the formation of a lignified cell layer and a layer of cells that mediates the separation through secretion of cell-wall-degrading enzymes (Petersen et al., 1996, Spence et al., 1996).

The main tissues that compose the developing fruit are schematically outlined in Figure 1D–1G, with the lignifying and separation layer together forming the VM. INDEHISCENT (IND) is a bHLH-type transcription factor required for VM development (Liljegren et al., 2004). One of the functions of IND is to establish an auxin minimum at the VM prior to dehiscence (Sorefan et al., 2009). This is achieved at least in part by repressing the PINOID (PID) gene, which encodes a protein kinase involved in polar localization of PIN auxin transporter. The auxin minimum is located at the VM and was shown to be functionally important for dehiscence (Figure 1C), (Sorefan et al., 2009). It is clear that this functional auxin minimum requires an active process (rather than being the inevitable valley of low concentrations that has to exist between two regions containing maxima), because (i) it develops in a temporally regulated fashion, unlinked to specific auxin accumulation in the flanking regions; (ii) it is of a striking qualitative nature, with much lower auxin levels seen in the very narrow tissue region of the VM, but running longitudinally over the whole silique; and (iii) the quantitative and qualitative drop in auxin are directly linked to fruit maturation and dehiscence. It is thus a developmentally instructive minimum, which in that sense shares features with the auxin minimum that can be found at the transition zone in the root apical meristem, responsible for triggering the switch from dividing to elongating and differentiating cells. Also in the root, a qualitative and substantial drop in auxin can be found within a transversally confined region with developmental relevance; a pattern that is moreover spatio-temporally regulated and also cannot be explained as simply a manifestation resulting from neighboring maxima (Di Mambro et al., 2017). Here, we apply a systems biology approach to ask how the fruit is able to sustain the characteristic low auxin concentrations in tissues that are only two to three cell files wide but longitudinally run over the entire fruit length. Such a quasi-one-dimensional auxin minimum directly flanked by plateaus of higher auxin concentrations might be expected to readily homogenize with the neighboring tissue (Han et al., 2014), thus abolishing the minimum, except when active transport processes prevent this from happening. We therefore question what kind of auxin transporter patterns are required to form this auxin profile, what processes establish and maintain the minimum, and what this implies for the auxin fluxes through the tissue. Note that within this work, we do not study the dynamical auto-organization of transporter expression and polarity that would lead to the transporter patterns themselves.

We therefore combine computational and experimental approaches to identify the conditions required to maintain such a distribution, and, by studying the flux patterns that ensue, generate novel predictions regarding plausible auxin transporter functionalities underpinning this process within a specific tissue context. Using computational modeling, we show that this system requires apolar auxin efflux in the VM cells combined with influx within the surrounding tissue to produce an auxin minimum at the VMs. Moreover, based on auxin flux predictions *in silico* and by perturbing auxin flux *in planta*, we show that directed efflux at the VM provides the primary driver for producing the VM auxin minimum. Interestingly, this process is fundamentally different from how minima were originally predicted to arise within the context of canalization models (Mitchison, 1980b, Mitchison, 1981).

## Results

Flower and fruit development in *Arabidopsis thaliana* has been intensely studied over decades. To facilitate this work, its flower development was divided into a series of stages based on the chronological occurrence of specific developmental events from the initial emergence of floral meristem to the final dispersal of the seeds (Smyth et al., 1990, Roeder and Yanofsky, 2006). In this work, we consider events that take place in development through stages 15–17b when fruit elongation takes place (15/16), the fruit fully matures, and the VM differentiates into a dehiscence zone (17b).

First, we used computational modeling to assess the auxin patterns that arise when taking into account known data regarding the tissue organization of the fruit and the polar localization and expression levels of the auxin transporters. These auxin transporter localization and expression patterns are not considered to alter during the course of the simulations. To this end, we captured the outermost epidermis of the fruit, with its different cell types, in a multicellular modeling description at a subcellular resolution (Figure 1D–1H). We display simulation results in a 2D flattened-out form (Figure 1G). In this description, we assumed an influx term of auxin from the topmost cells, representing auxin derived from the style tissue, capturing local apical auxin biosynthesis (Eklund et al., 2010, Kuusk et al., 2002, Cheng et al., 2006). In addition, low levels of biosynthesis and decay of auxin were homogeneously distributed over the whole tissue within all cells (see Methods and Supplemental Information for detailed model description, and Supplemental Tables 1 and 2 for parameter values). Auxin dynamics then result from those reaction terms, combined with diffusion in the cell wall and in the cytoplasm. We also took into account transport across cell membranes due to background influx and very low efflux permeability rates (reflecting the chemiosmotic nature of auxin transport), together with augmented influx and efflux contributed by the AUX1/LAXs and PINs (see Figure 1H, Methods, and Supplemental Information). Such an approach allows us to quantitatively distinguish between influx- and efflux-mediated contributions within this modeling framework. The model describes the characteristic polarity of these cells, without simulating the underlying dynamics of the intracellular partitioning itself (Abley et al., 2013, Grieneisen et al., 2013). Auxin was assumed to freely leave the fruit organ basally, capturing the connectedness of the fruit to the rest of the plant.

**Figure 2:**
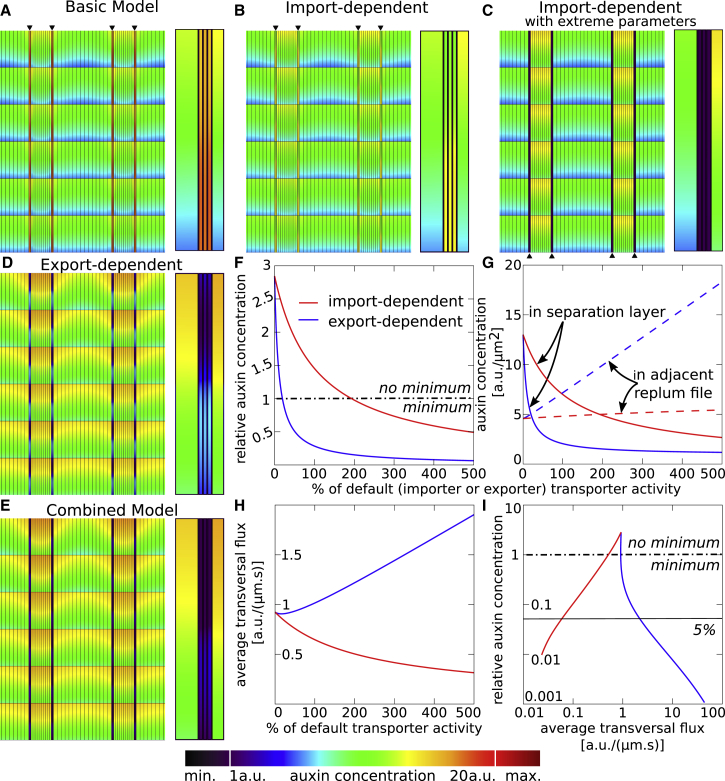
Minimal Requirements for Auxin Minimum at the VM. **(A)** Basic model, based on currently published transporter expression, predicts an auxin maximum, rather than minimum, in the VM; right inset shows details of minimum, by showing a magnified, one-cell-high portion of the left VM, including an outer adjacent valve cell and an inner adjacent replum cell. **(B and C)** Import-dependent model shows that when all tissues except the VM have augmented influx activity, the minimum does not form under reasonable auxin importer transporter rates; as seen through right inset of magnified VM **(B)**. In contrast, the minimum is only established under very high transporter rates (background influx set at $P_{IAAH}$ = 5 μm/s; augmented influx at $P_{LAX1}$ = 100 μm/s) **(C)**, with right inset showing corresponding VM minimum. **(D)** Export-dependent model reveals that the default rate of apolar efflux within the VM is sufficient to create an auxin minimum, as seen in detail in the right inset. **(E)** A combination of apolarly localized efflux transporters and VM-specific lack of influx transporters (combined model) strengthens the auxin minimum, as seen in detail in the right inset. **(F and G)** Both strengthening apolar exporters in the VM (blue line, export-dependent case) and apolar importers in the valve and replum (red line, import-dependent case) lead to a decrease in the ratio between the auxin concentration in the separation layer and in the bordering replum cell **(F)**, as well as a decrease in the absolute auxin levels within the separation layer (**G**, solid lines). Auxin levels in the replum, however, increase with increasing transporter strength in the export-dependent model, but only marginally depend on the transporter strength in the import-dependent model (**G**, dashed lines). **(H)** Effect of those transporters on the total transversal fluxes crossing the VM (i.e., perpendicular to the VM). The *x* axes in **(F–H)** indicate the relative strength of either the VM-specific exporter (blue), or the augmented importer in the valve and replum (red), as a percentage of the default transport rates. **(I)** The fluxes crossing the VM transversally plotted against the VM minimum (as calculated in **F**), on a log-log scale. Details of parallel fluxes are shown in Supplemental Figure 2, and the description of average flux calculations is given in the Supplemental Information. Dashed-dotted lines indicate where the auxin level in the VM is equal to the surrounding tissue, i.e., below which an auxin minimum is formed; thin line indicates where the auxin level in the VM is 5% of the level in the surrounding tissue. Color bar indicates auxin concentrations in **(A–E)**. Arrowheads in **(A–C)** indicate position of VM.

Two different reporters have been used to visualize the auxin minimum in the *Arabidopsis* fruit, namely DR5::GFP (Sorefan et al., 2009) and DII-VENUS (van Gelderen et al., 2016). While the DR5 reporter monitors auxin response gene expression, DII-VENUS is considered an auxin sensor and is degraded in the presence of auxin in a TIR1/AFB-dependent manner (Brunoud et al., 2012). Hence, both report auxin signaling rather than auxin levels, and we therefore define an auxin minimum here as a region of low auxin signaling. The auxin minimum at the VM is most evident at developmental stage 17b (Figure 1C) (Sorefan et al., 2009, van Gelderen et al., 2016). At this stage, published distributions of PIN transporters have only reported the presence of PIN3 and only in the valve and replum cells, where it is localized basally (Sorefan et al., 2009, van Gelderen et al., 2016). Reflecting this consensus, we firstly considered the patterns that emerge when PIN3 is only expressed in the replum and the valve tissues, using the intensity and localization as observed, and without taking any additional importers into account. This was done by attributing higher permeability to specific polar cell membrane domains (Supplemental Figure 1), promoting auxin efflux. We call this setting the basic model (see Methods and Supplemental Information, and Supplemental Tables 1 and 2 for parameter values). Rather than becoming depleted from the VM, the resultant patterns showed that auxin would instead accumulate in both the separation and lignifying layers of the VM (Figure 2A). This indicates that currently reported transporter distributions are not able to account for the auxin minimum and raises the question what is needed to generate the observed auxin minimum.

### Minimal Requirements for VM Minimum: Two Basic Modes

To determine the dynamic activities involved in auxin distribution at the VM, we next sought to identify the basic necessary conditions that could yield an auxin minimum in the VM in the most parsimonious manner. Firstly, the large differences in auxin concentration over a narrow region, within a tissue that also displays apical-basal auxin fluxes and large diffusion rates, excludes as a possible explanation classical reaction-diffusion models of morphogen patterning, such as those based upon production and decay. To confer this, we simulated such a production-breakdown mechanism, removing any differences in transporters between cell types (see Table 1), instead limiting auxin production to replum and valve and confining breakdown to the VM. These reaction-diffusion simulations show that for a noticeable auxin minimum to be formed and confined to the VM, the auxin breakdown in the VM has to be very fast, in fact, at least eight orders of magnitude larger than what is considered biologically reasonable (Supplemental Figure 3). Such an extremely fast breakdown would preclude any relevant non-local phytohormone signaling (Grieneisen et al., 2012), rendering a production-degradation mechanism non-viable.

**Table 1:** Relative Auxin Importer and Exporter Strengths at the Different Facets along the Plasma Membrane, as Used in the Conceptual Models.

	Upper-outer	Upper-inner	Inner-upper	Inner-lower	Lower-inner	Lower-outer	Outer-lower	Outer-upper
**Basic Model**								

PIN3								
Replum	0	0	0	0.5	1	1	0.5	0
Valve	0	0	0	0.5	1	1	0.5	0

**Import-Dependent**								

PIN3								
Replum	0	0	0	0.5	1	1	0.5	0
Valve	0	0	0	0.5	1	1	0.5	0
LAX1								
Replum	1	1	1	1	1	1	1	1
Valve	1	1	1	1	1	1	1	1

**Export-Dependent**								

PIN3								
Replum	0	0	0	0.5	1	1	0.5	0
Separation layer	1	1	1	1	1	1	1	1
Lignifying layer	1	1	1	1	1	1	1	1
Valve	0	0	0	0.5	1	1	0.5	0

**Combined**								

PIN3								
Replum	0	0	0	0.5	1	1	0.5	0
Separation layer	1	1	1	1	1	1	1	1
Lignifying layer	1	1	1	1	1	1	1	1
Valve	0	0	0	0.5	1	1	0.5	0
LAX1								
Replum	1	1	1	1	1	1	1	1
Valve	1	1	1	1	1	1	1	1

**Production Decay**								

PIN3								
Replum	0	0	0	0.5	1	1	0.5	0
Separation layer	0	0	0	0.5	1	1	0.5	0
Lignifying layer	0	0	0	0.5	1	1	0.5	0
Valve	0	0	0	0.5	1	1	0.5	0

The facets are as depicted in Supplemental Figure 1, denoted as major-minor orientation. Values are only given for cell types that contain the specific transporter.

In contrast, two other processes can be envisioned, which are instead based on modifications of polar auxin transport. The first possibility, the import-dependent model, is that all tissues, except for the VM, retain auxin through enhanced import, thereby sequestering auxin away from the VM; the second, the export-dependent model, is that the VM itself depletes auxin through active export as previously proposed (Sorefan et al., 2009). We tested this import-dependent model through simulations that assume all tissues, except for the VM, are endowed with high levels of apolarly localized influx transporters (see Table 2 for transporter expression patterns). We found that at typical parameter values used for auxin influx (Grieneisen et al., 2012, Di Mambro et al., 2017), only a meagre reduction in auxin at the VM occurs, compared with the basic model (compare Figure 2A and 2B). In fact, auxin levels are still higher in the VM than within the other tissues (Figure 2B). To generate an auxin minimum within the VM in an import-dependent manner (Figure 2C), import permeability via the AUX1/LAX family importers needs to be at least 20 times larger than the background influx permeability. Moreover, even when these differences in permeability are extremely large (for example, more than 1000-fold), the auxin minimum still never becomes less than half the level found in the surrounding tissues. In contrast, when we ran the export-dependent model in the *in silico* fruit by introducing apolar PINs in the VM at reasonable permeability rates (such as previously reported, Grieneisen et al., 2012), we observed an immediate, striking drop in the auxin concentrations within the VM (Figure 2D). Thus, our model suggests that actively exporting auxin from the VM is a more efficient mechanism to establish an auxin minimum. Combining both scenarios (combined model), now using reasonable permeability values for the AUX1/LAX-driven auxin influx, synergistically generated an even more pronounced auxin minimum at the VM (Figure 2E). To quantitatively explore the difference between these models, we assessed the effect of the strength of the apolar exporter in the VM or the strength of the apolar importer in the valve and replum by calculating the resultant ratio between the auxin concentration in the separation layer and in the bordering replum cell (Figure 2F). This ratio determines the percentage auxin decrease within the VM and provides a good assessment of the magnitude of the auxin minimum. Moreover, we concomitantly analyzed the absolute auxin levels in both tissue types (Figure 2G). By performing a large parameter sweep, we found that a low level of apolar efflux activity in the VM (at 10% of the default permeability rate) is sufficient to generate a minimum. In contrast, only very strongly augmented import in the surrounding tissues (more than 200% of the default permeability rate) is able to generate an auxin minimum. Moreover, in the export-dependent model, apolar exporters in the VM acting at a strength of 70%, compared with the export permeability in the other tissues, yields a very well-defined minimum with a depth that the import-dependent model is unable to generate for any level of augmented import permeability (Figure 2F). This is partly because the import-based model is unable to substantially raise the auxin levels in the replum, whereas increasing efflux activity gave rise to a linear increase in the absolute concentrations in the replum (Figure 2G). Moreover, the export-dependent model greatly reduced the levels in the separation layer in a way that was much more responsive to alterations in its transporter activity than found for the import-dependent model (Figure 2G). This sensitivity analysis shows that local transport modifications in the VM, under the efflux-dependent scenario, can have spatially long-reaching effects in other tissues.

**Table 2:** Relative Auxin Importer and Exporter Strengths at the Different Facets along the Plasma Membrane, as Used in the Detailed Models.

	Upper-outer	Upper-inner	Inner-upper	Inner-lower	Lower-inner	Lower-outer	Outer-lower	Outer-upper
**Stage 17b**								

PIN3								
Replum	0	0	0	0	0.8	0.8	0	0
Separation layer	0.2	0.2	0.2	0.2	0.2	0.2	0.2	0.2
Lignifying layer	0.2	0.2	0.2	0.2	0.2	0.2	0.2	0.2
Valve	0	0	0	0.4	1	1	0.4	0
PIN7								
Valve	0	0	0	0	1	1	0	0
LAX1								
Replum	1	1	1	1	1	1	1	1
Valve	0.2	0.2	0.2	0.2	0.2	0.2	0.2	0.2

**Stage 16**								

PIN3								
Replum	0.8	0.8	0.4	0.4	0.8	0.8	0.4	0.4
Separation layer	0.2	0.2	0.2	0.2	0.2	0.2	0.2	0.2
Lignifying layer	0.2	0.2	0.2	0.2	0.2	0.2	0.2	0.2
Valve	0	0	0	0.4	1	1	0.4	0
PIN7								
Replum	0	0	0	0	0.1	0.1	0	0
Valve	0	0	0.2	0.2	1	1	0.2	0.2
LAX1								
Replum	1	1	1	1	1	1	1	1
Valve	0.8	0.8	0.8	0.8	0.8	0.8	0.8	0.8

**Stage 15**								

PIN3								
Replum	0.8	0.8	0.4	0.4	0.8	0.8	0.4	0.4
Valve	0	0	0	0.4	1	1	0.4	0
PIN7								
Replum	0	0	0	0	0.1	0.1	0	0
Valve	0	0	0.2	0.2	1	1	0.2	0.2
LAX1								
Replum	1	1	1	1	1	1	1	1
Valve	0.8	0.8	0.8	0.8	0.8	0.8	0.8	0.8

**Figure 3:**
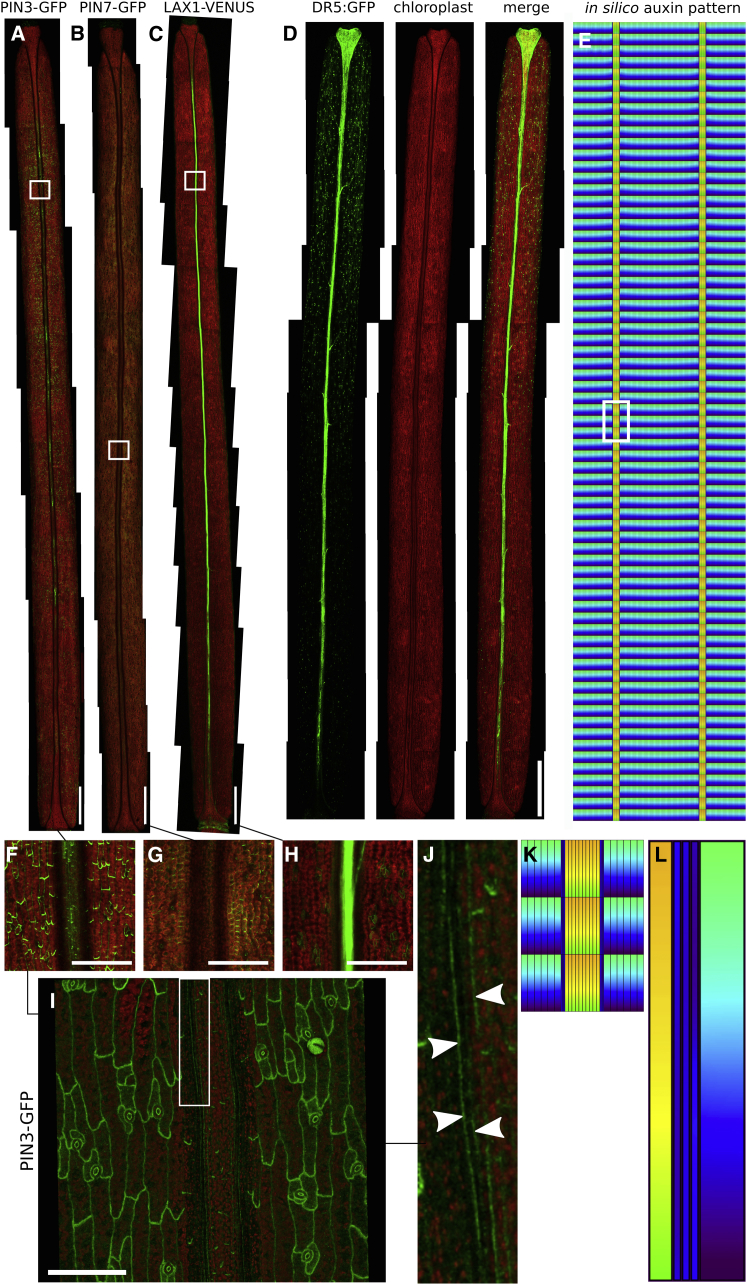
Detailed Analysis on Actual Transporter Localizations at Stage 17b **(A)***PIN3::PIN3-GFP*. **(B)***PIN7::PIN7-GFP*. **(C)***LAX1::LAX1-VENUS*. **(D)***DR5::GFP*. **(E)** Simulation using imaged transporter localization and levels at stage 17b and tissue size and layout of that stage presents minimum at VM and elevated levels in the replum, in agreement with the experimentally observed auxin-signaling pattern. **(F–H)** Detailed insets from **(A–C)**, as indicated. **(I and J) (I)** Detailed image showing apolar PIN3 localization, with **(J)** showing further magnification of PIN3 localization within a VM region indicated by a white rectangle in **(I)**. Arrowheads indicate the position of lateral PIN3-GFP in the VM. **(K)** Inset from **(E)**, as indicated. **(L)** Magnified right portion of the VM, indicating in detail the auxin minimum in **(K)**. See Supplemental Figure 4 for further supporting experimental images. Scale bars: 1 mm for **(A–D)**; 200 μm for **(F–H)**; 100 μm for **(I)**. Color coding of auxin levels as indicated in Figure 2.

The facets are as depicted in Supplemental Figure 1, denoted as major-minor orientation. Values are only given for cell types that contain the specific transporter. Matrixes for stage 17b and 16 are semi-quantitative approximations based upon careful (human) assessment of the microscopy images, as depicted in Figures 3 and 5, respectively.

### Experimental Confirmation of PIN Localization in the VM

Our simulations strongly suggested that apolar PIN localization in the VM cells is necessary and sufficient to generate an auxin minimum in the VM. This is in agreement with the findings of Sorefan et al. (2009) who demonstrated that the VM-specific transcription factor INDEHISCENT (IND) is required for the minimum to form and is a repressor of the *PINOID (PID)* gene. *PID* encodes a protein kinase involved in the regulation of PIN polarization (Benjamins et al., 2001, Friml et al., 2004); however, whereas ectopically polarized PIN3-GFP was detected across all cell files in *ind* mutant fruits, no signal could be detected at the VM of wild-type fruits by the confocal microscopy setup used (Sorefan et al., 2009).

Therefore, to further test the prediction that enhanced auxin efflux occurs at the VM, we analyzed reporters of PIN expression and localization during the late stages of fruit development. Confocal imaging confirmed previous observations that *PIN3* is expressed in the valves of the *Arabidopsis* fruit at stage 17b and that the PIN3-GFP protein is primarily localized at the basal side of the cells (Figure 3A and 3F) (Sorefan et al., 2009). Previous research suggested the existence of apolarly localized PIN efflux carriers in the VM, but was unable to detect this (Sorefan et al., 2009). Indeed, visualizing confocal Z stacks in 3D using VolViewer software (Lee et al., 2006) revealed low *PIN3::PIN3-GFP* expression at the VM with apolar localization of the PIN3-GFP protein (Figure 3I and 3J; Supplemental Video 1 for a clear 3D view). These data suggest that PIN3 contributes to auxin efflux from the VM into the surrounding tissues. Also a *PIN7::PIN7-GFP* reporter (Blilou et al., 2005) was found to be expressed at this stage, specifically in the valve, albeit at lower levels than PIN3. No expression, however, could be detected in the VM (Figure 3B and 3G). Finally, a *LAX1::LAX1-VENUS* reporter was found to be also expressed at this stage, in the valve and at particularly high levels in the replum, while—in agreement with the computational model—expression was restricted from the VM domain (Figure 3C and 3H). Interestingly, the LAX1 pattern mimics the expression pattern of the auxin-signaling reporter *DR5::GFP*, which also presents a high signal in the replum at this stage (Figure 3D). In addition to these reporters for *PIN3*, *PIN7*, and *LAX1*, we tested reporters for *PIN1*, *PIN4*, *AUX1*, *LAX2*, and *LAX3*, but were unable to detect their expression in the fruit at the developmental stages studied in this manuscript.

Supplemental Video 1. PIN3 Distributions in the VM 3D imaging reveals PIN3 apolar localization to the cells of the VM

We next questioned whether the observed PIN3 and LAX1 levels and localization would also quantitatively be sufficient for generating the observed VM auxin minimum and patterning (Figures 3D and 1C). To answer this, we translated the experimentally observed fluorescence values of PIN3, PIN7, and LAX1 (Figure 3A–3C), which were captured using a fixed laser intensity into values ranging from 0 to 1, where 0 corresponds to no enhanced permeability and 1 corresponds to maximum transport permeability rates, for each combination of transporter, cellular polar domain (Supplemental Figure 1), and tissue (Figure 1E). Table 2 provides all the image-derived normalized permeability rates. For simplicity, we assumed a linear relationship between fluorescence levels and transport strength, ignoring potential saturation in transport, non-linearity in the relationship between fluorescence and protein levels, and transporter post-processing affecting transport strength. Highest overall observed fluorescence levels for each individual transporter were used to normalize the permeability rates. For the spatial simulation, we measured the typical width and height of individual cells belonging to a specific cell type from the experimental images, as well as the number of cell rows and cell files within each tissue. We then ran *in silico* simulations of auxin dynamics, incorporating the experimentally derived transporter patterns and intensities with the experimentally derived fruit layout (Figure 3E). Under these settings, the simulation resulted in an auxin pattern with a minimum matching that observed experimentally (Figure 3K and 3L). Furthermore, the simulation captured other aspects of the observed auxin pattern, in particular the significantly higher auxin levels in the replum compared with the valve. Note that the transporter quantification was done independently, before running the simulations, and not modified *a posteriori*, to prevent bias. We therefore conclude that the observed PIN3 levels, in conjunction with LAX1 levels, can account for the auxin minimum.

### The VM: A Flux Passage or a Flux Barrier?

The models described above show that the VM minimum can be established through tissue-specific expression patterns of either influx carriers at high activity levels (Figure 2C) or efflux carriers (Figure 2D), or through an appropriate interplay of both (Figure 2E). When we subsequently sought to test these processes experimentally, we obtained evidence supporting both. Indeed, realistic simulations (Figure 3E) revealed that the experimentally observed transporter distributions are able to generate the auxin minimum as well (Figure 3K and 3L). It is difficult, however, to assess only by means of GFP transporter expression patterns what the relative contribution of the influx and efflux carriers are to the resultant pattern, i.e., which effective process/mechanism is predominantly being deployed. Moreover, single transporter mutants are notoriously difficult to interpret, due to redundancy and compensation (Blilou et al., 2005, Grieneisen and Scheres, 2009), rendering a systems biology approach to the problem necessary. Our initial parameter sweep suggested that an efflux-dependent process is primarily involved in producing the VM minimum, while the importer-based process only makes a minor contribution (Figure 2F and 2I). A putative candidate for the implementation of the efflux-dependent model is via PINs. However, an important confounding factor for the modeling is that fluorescence is not a direct indication of the actual permeability rate and a full-strength PIN3 permeability might therefore be very different from a full-strength LAX1 permeability. Therefore, even though our analysis (Figure 2F and 2G) showed that to produce a comparable auxin minimum, much higher augmented influx permeability rates are required (for example, via LAX1) than localized efflux rates (possibly, through PIN3), neither the experiments nor simulations presented can convincingly conclude that the efflux-based process is indeed the predominant process by which the minimum is established. Moreover, it is possible that other transporters, such as ABCBs (ATP-binding cassette transporters of the B subfamily) (Bandyopadhyay et al., 2007, Geisler et al., 2017), might be functionally present as well. In short, despite our finding that PIN3 localization at the VM is supportive of the active efflux-dependent model, the LAX1 expression observed in the surrounding tissues likewise supports the alternative hypothesis that sequestering auxin by the other tissues could be a driving process for generating the minimum. As both scenarios generate similar outcomes on the level of auxin distributions under appropriate parameter conditions, we sought an additional and alternative observable to distinguish between them.

We found that although both processes are able to generate qualitatively similar steady state auxin patterns, the auxin fluxes underlying them are both qualitatively and quantitatively very different (Figure 2F and 2H; Supplemental Figure 3). At the location of the auxin minimum, in the VM, the import-based process shows negligible auxin throughput over and along the VM (Supplemental Figure 3L and 3Q). In contrast, while still maintaining very low auxin levels at the VM, the efflux-based scenario yields considerably higher fluxes over the VM, transversally connecting the valve to the replum (Supplemental Figure 3M and 3R) through perpendicular fluxes across these cell files. Also the combined model would predict such transversal flows (Supplemental Figure 3N and 3S). To further quantify these patterns, we calculated how the fluxes through the VM depend on the strength of the apolar exporter in the VM or on the strength of the apolar importer in the valve and replum. Interestingly, while the import-dependent scenario presents decreasing overall fluxes with decreasing auxin levels (Supplemental Figure 2C, for total flux magnitudes) with negligible and decreasing transversal fluxes across the VM, as would be the intuitive expectation, the efflux-dependent scenario in contrast presents increasing fluxes across (i.e., perpendicular to) the VM (Figure 2H) with decreasing auxin levels within the VM (Figure 2F, 2G and Supplemental Figure 2A and 2C). In short, the export-dependent scenario, by removing in all directions any auxin that enters these cells, generates an effective flux-passage-type process, allowing auxin to cross these files transversally. In contrast, the import-dependent case, preventing the entrance of auxin in the first place, generates a flux-barrier-type process, although a small level of parallel fluxes does linger (Supplemental Figure 2B). Plotting the relative strength of the minimum against the total fluxes that cross the minimum perpendicularly (Figure 2I) further illustrates this behavior: for a quantitatively similar minimum (e.g., of 5%, as indicated in the figure), the flux-barrier and the flux-passage processes present a 100-fold difference in regard to the resultant transversal auxin fluxes.

Note that also canalization models, as first proposed by Mitchison (1980a), predicted high auxin fluxes along veins containing lower auxin concentrations than the surrounding tissue. These models were based on the premise that fluxes effectively self-enhance themselves, triggering thereby a self-organized vasculature patterning. Subsequent experimental observations, however, showed that leaf veins actually have high auxin concentrations (Mattsson et al., 2003). Moreover, the nature of the fluxes as presented by the paradigmatic canalization mechanism are very different from the ones predicted by the flux-passage mechanism we report here. In our model, high fluxes occur across the quasi-1D structure of the VM and actually increase as transporter activity parameters strengthen the minimum; in contrast, the fluxes occurring along the VM do not increase as the minimum deepens (Supplemental Figure 2A and 2B). This contrasts to the behavior resulting from canalization models, where there are negligible fluxes crossing the veins perpendicularly, but large fluxes parallel to them.

While our computational model of auxin dynamics and patterning allows us to directly assess the underlying fluxes that result from any given transporter configuration, this is not possible experimentally. To overcome this, we hypothesized that physically blocking the auxin passage over the VM may allow for an indirect test of which flux mechanism is involved. In the case of a flux-passage process, blocking the VM should result in noticeable alterations in the auxin concentrations in the flanking tissues, while in the flux-barrier scenario, such a physical obstruction should only result in marginal differences in the auxin distribution within flanking tissues. Such differences, or lack of differences, in auxin concentration should be experimentally trackable through the DR5 auxin-signaling reporter and would allow us to assess whether the flux-passage process (PIN-mediated efflux from the VM) or flux-barrier process (LAX-mediated influx from surrounding tissue) predominates.

**Figure 4:**
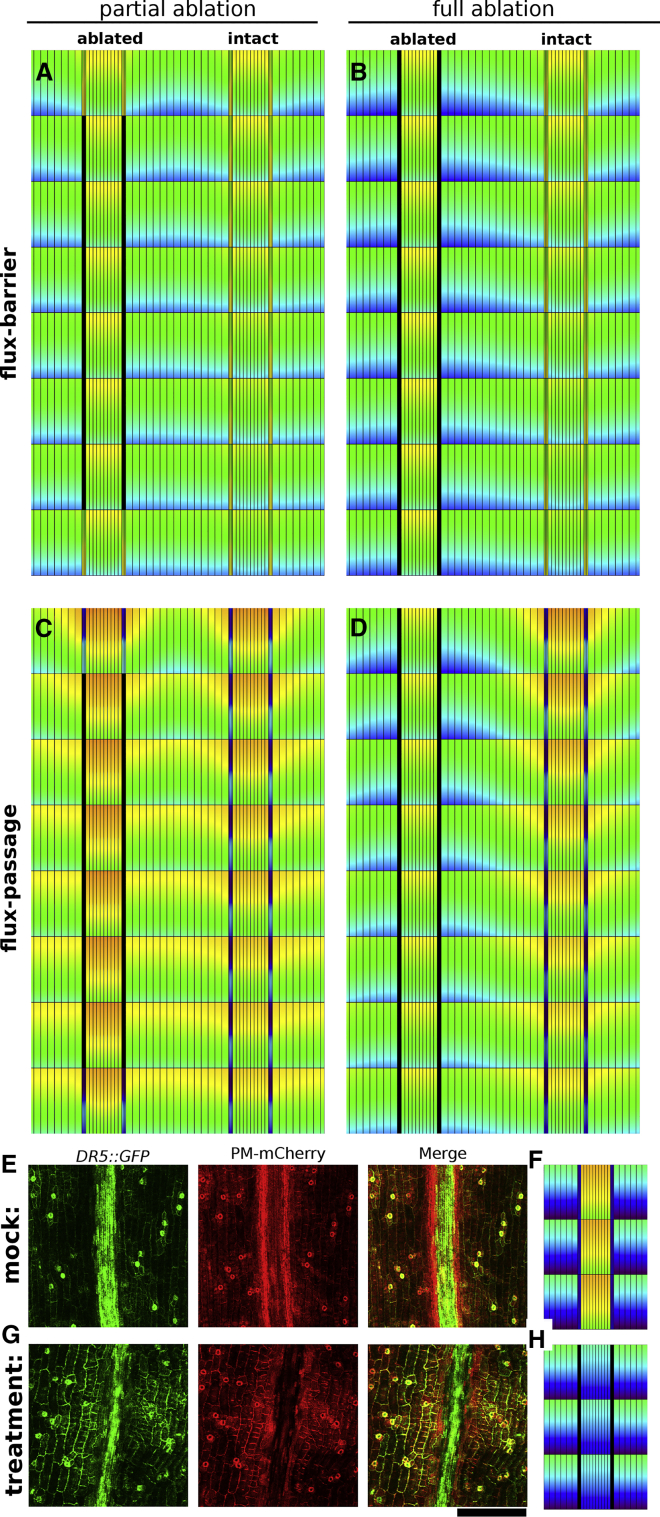
Interfering with the Auxin Fluxes through the VM. **(A and B)** Modeling predicts that if the auxin minimum is solely due to lack of augmented influx activity in the VM, then partly **(A)** or fully **(B)** ablating the VM only slightly changes the auxin levels in the valve and replum. **(C and D)** In contrast, if the auxin minimum were due to apolar PIN3 in the VM, then partly **(C)** or fully **(D)** ablating the VM strongly affects the auxin levels in those tissues. To better illustrate the impact, only the VMs flanking one of the repla are ablated. **(E and G)** DR5:GFP in control treatment **(E)** and after DEX-induced VM ablation **(G)**. **(F and H)** Predicted auxin pattern using the full model for stage 17b **(F)** after VM ablation **(H)**. Scale bar: 200 μm for **(E and G)**. Color coding of auxin levels as indicated in Figure 2.

We first explored what effects ablating portions of the VM would yield in both contrasting scenarios. However, introducing a small *in silico* ablation at the VM in either the influx-dependent or efflux-dependent model (see Supplemental Information for modeling implementation) did not generate any noticeable changes in the auxin distributions (Supplemental Figure 5F–5I), despite them displaying distinct flux-barrier and flux-passage processes, respectively. Further *in silico* ablations revealed that only larger extents of VM obstruction cause noticeable changes in the auxin pattern (Figure 4C), with the model displaying the flux-passage process showing only a modest increase in the replum upon ablation, while the alternative model, the flux-barrier process, is unaffected (compare Figure 4A with 4C). Note that we assume for the *in silico* ablations that all domains maintain fixed PIN distributions and intensity, both before and after ablation. Hence, dynamical changes in auxin profiles and flux patterns that can be observed are solely due to VM ablation. (Although transporter reorientations might occur at longer timescales after ablation, we do not consider them within this modeling framework.)

We therefore realized that to experimentally verify the predicted distinction between the processes, only partially ablating the VM might not be sufficient. Indeed, small ablation extensions resulted only in minor effects (Supplemental Figure 5, Methods, and Supplemental Information for details). However, when ablating larger extents, changes in the auxin profile did occur, especially close to the VM, but in a very inconsistent and irreproducible manner (Supplemental Figure 5, and Dryad Repository for additional images). Although some ablation experiments provided support for the hypothesis that underlying fluxes are at play passing through the VM's auxin minimum, it also revealed the sensitivity of these experiments to the extent of the ablated region, and to possible wound damage responses in the surrounding tissues, given the large extent of tissue being laser ablated. Therefore, we sought an alternative method to test the model predictions.

From the simulations, it also became clear that even if a small region of the VM is kept intact after ablation, this will be sufficient to redistribute part of the auxin that would otherwise have accumulated alongside the VM, thereby obscuring the effect of the obstruction (compare for example, Figure 4C and 4D). In order to achieve the blockage of the whole VM, we therefore developed an inducible BARNASE system, for expression specifically at the VM. The *BARNASE* gene encodes a powerful toxin, which upon expression will stimulate cell death. In order to keep its toxic effect confined to the cell expressing it, the *BARNASE* gene is fused to its inhibitor, BARSTAR (Beals and Goldberg, 1997). In the two-component system employed here, the VM-specific *IND* promoter drives the expression of the LhGR transcription factor (LhG4 transcription factor fused to the Glucocorticoid Receptor), while the *BARNASE-BARSTAR* gene is under control of the pOp promoter recognized by the LhGR protein. Upon treatment by dexamethasone (DEX), the LhGR protein produced in the VM will enter the nucleus and induce expression of the toxin. The effect of inducing the *IND>GR>BARNASE-BARSTAR* system can be seen after 3–9 days of treatment (see Methods), with scanning electron micrographs showing the damaged VM cells (Supplemental Figure 7). Importantly, the effect is highly local, as the wound-induced reporter line *WIND1::GUS* is only expressed in the VM cells (Supplemental Figure 7). The genetic ablation of the VM cells was verified using an mCherry marker for the plasma membrane (PM) (Nelson et al., 2007). Without DEX treatment, this line clearly marks the VM cells, whereas the PM of these cells has disappeared in the presence of DEX (Figure 4E and 4G). Therefore, this system allows us to induce cell death of the entire VM in a temporally controlled manner, without causing collateral damage to the surrounding tissues. In contrast to the control treatment (Figure 4E), such a chemically induced obstruction of the VM, at stage 17b, resulted in a consistent and significant drop of auxin in the replum cells directly flanking the VM, as well as a rise in auxin in the valve cells (Figure 4G). The observed pattern closely corresponded to the pattern predicted by modeling full ablation in the situation in which differences in efflux carriers yield the flux-passage process (Figure 4D, with the VMs flanking the left replum being ablated while the ones flanking the right replum stay intact, for straightforward comparison). In our ablation simulations, only cell death (total impermeability) of the VM tissue was taken into account, using the assumption that no changes in transporter intensity and localization in the adjacent tissues occurred. To establish if this is a reasonable assumption, we crossed PIN3:PIN3-GFP with the IND>RG>BARNASE-BARSTAR line to be able to observe PIN3 intensity and localization after chemical ablation of the VM at 9 days after the initialization of DEX treatment. No noticeable changes in PIN3 intensity or localization were observed in the replum or valve after chemical ablation of the VM, supporting the modeling assumption of unaltered transporter patterning (Supplemental Figure 8). The dynamic effect of VM ablation on auxin levels (Figure 4E–4H) therefore indicates that transversal auxin fluxes are taking place at the VM at stage 17b even though the auxin levels themselves are low. Taken together, these results predict that the auxin minimum predominantly involves active efflux across the VM, with lower influx possibly contributing to the depth of the minimum. In addition, our combined modeling and experimental data suggest that differences in transporter patterns within the valve and replum underlie the constant flux over the VM.

The auxin minimum at the VM plays a well-defined developmental role in maintaining the position and regulating the temporal timing of dehiscence. We therefore next analyzed the temporal regulation of the minimum.

### Efflux: Digging Deeper into the Minimum

**Figure 5:**
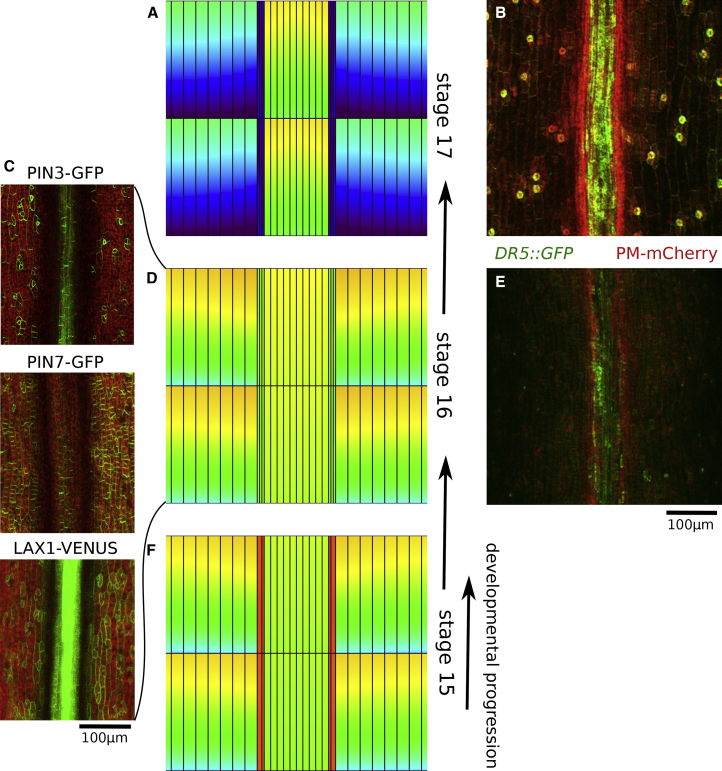
Temporal Development of the VM Minimum. **(A and B)** Auxin patterning as predicted by the model **(A)** and experimentally observed **(B)** during stage 17b. **(C–E) (C)** PIN3:PIN3-GFP (top); PIN7:PIN7-GFP (middle); LAX1:LAX1-VENUS (bottom) at stage 16, with **(D and E)** auxin patterns as predicted by the model **(D)** and experimentally observed **(E)**. **(F)** Predicted auxin patterns using the full model for stage 15. **(A, D, and F)** illustrate the formation of the auxin minimum in the VM and build-up of auxin in the replum over time. Scale bars as indicated. Color coding of auxin levels as indicated in Figure 2.

The results described above from experimentally induced tissue perturbations support the notion that auxin efflux at the VM plays a central role in the auxin minimum formation. Based on imaging, PIN3 may be a key factor in this process, although we cannot exclude the action of yet unknown auxin exporters in this region. Thus far, our analysis was confined to a single developmental stage, 17b (Figure 5A and 5B). If the expression patterns that we took into account to explain that specific developmental time points are indeed determining the auxin and flux patterns, we would expect that observed differences in transporter expression at other stages should roughly correlate with the predicted auxin patterns at those different stages as well. With this in mind, we extended our analysis to an earlier developmental stage, 16, and generated a detailed map of relative permeability rates based on PIN3, PIN7, and LAX1 localization and intensity patterns (Figure 5C and Supplemental Figure 9, and Table 2). The main developmental difference considered between stages 17b and 16 is that at stage 16, PIN3 is more abundant in the replum, while PIN7 is slightly more abundant in the valve, as is LAX1. Running the model generated auxin distributions that matched observed auxin-signaling patterns of DR5::GFP (Figure 5D and 5E). Notably, the auxin minimum in the early stages was not as prominent as during later stages (when comparing stage 16 and 17b) (Figure 5A, 5D, 5B, and 5E), as reported previously (Sorefan et al., 2009, van Gelderen et al., 2016).

We extrapolated these insights to an even earlier developmental time point, around stages 14–15, based upon recently published data regarding this stage (van Gelderen et al., 2016). We captured this earlier stage by reducing the strength of PIN3 in the VM. Moreover, the number of cell files was reduced taking into account that the VM at stage 15 has not yet undergone the asymmetric cell division that specifies the separation and lignified cell layers (Supplemental Figure 6F and 6H) (Wu et al., 2006). The resultant auxin pattern for this early stage reveals higher auxin levels at the VM (Figure 5F) compared to stage 16 (Figure 5D). Thus, when VM-localized efflux is lower, auxin levels are predicted to be substantially higher, suggesting that the fruit ripens by gradually transiting from an initial auxin maximum at the VM to an auxin minimum later in development. These high auxin levels result from the fact that VM cells at early stages have a higher perimeter-to-area ratio, thereby gaining more auxin through the chemiosmotically biased influx (Supplemental Figure 6B and 6F), as well as from reduced auxin efflux activity (Supplemental Figure 6C and 6G). The sequence of DR5 expression patterns therefore matches the auxin pattern predicted by the model in a qualitatively temporal fashion (van Gelderen et al., 2016).

## Discussion

Auxin maxima have been studied extensively for a wide set of plant systems, and different modes of auxin maximum formation have been inferred (Grieneisen and Scheres, 2009, Grieneisen et al., 2012). The relative role of importers and exporters for auxin accumulation has been theoretically explored, showing that interplay between export and import can be critical (Kramer, 2004, Band et al., 2014). However, for such quantitative evaluation to be made, *in silico* plant models require the cell-wall compartment to be taken into account explicitly, as was done here. Only when this apoplastic compartment is explicitly treated can correct units of permeability for the exporters/importers be used, their functional role be separated, and hence their quantitative contributions be assessed (see also Kramer, 2004, Abley et al., 2013, el Showk et al., 2015). Hence, for a systems biology approach to reveal the processes of auxin transport underlying developmental patterning, one must treat the multi-scale nature of the transport phenomena, as done here.

Moreover, the mechanisms underlying instructive auxin minima, defined as regulated and biologically functional regions of lower auxin signaling, have not yet been mechanistically analyzed to the same extent as maxima. It was recently established that the regulated maintenance of an auxin minimum at the basal root meristem triggers cell differentiation (Di Mambro et al., 2017). This work provided a mechanistic and genetic explanation for how another important phytohormone, cytokinin, controls and positions this minimum through auxin degradation and alterations in polar auxin transport. In the root system, as a consequence of the continuous growth of the root tip accompanied by a continuous spatial translocation of the phytohormone patterning, cells rapidly transit through that auxin minimum, triggering swift auxin variations within each cell. In contrast, the auxin minimum in the silique is much more constrained, extending over the whole longitudinal dimension of the fruit while being only two to three cell files wide. Moreover, cells are confined to this pattern, suggesting a very different mechanism for auxin minimum formation and information processing. Indeed, here we show, combining spatial modeling and experiments in a systems biology approach, that there are two contrasting processes that could account for the stripes of auxin minimum observed at the VM of the *Arabidopsis* fruit. Those processes are indistinguishable at the level of relative auxin distributions. Our modeling indicates that the efflux-dependent “flux-passage” process, relying on active apolar exporter activity at the VM, is more robust than the “flux-barrier” process, which relies on augmented influx into the surrounding tissues. Moreover, the flux-passage process also appears more likely, given its convincing performance within a reasonable permeability range. Visualization of microscopy images in 3D confirmed that *PIN3* is co-expressed with the auxin minimum and that the PIN3 protein is apolarly localized, providing support for the flux-passage process. We therefore propose that a rise in PIN3 levels may be associated with a gradual transition of the fruit from presenting an auxin maximum in the VM at early stages (stage 15; van Gelderen et al., 2016) to displaying an actual minimum at stage 17b. This process is correlated with the ripening of the fruit.

Interestingly, we found that the auxin minimum in the VM at stage 17b is obtained and maintained despite significant transversal fluxes across that tissue. Obstructing the VM tissue both *in silico* and *in vivo* over its entire longitudinal extension resulted in similar changes in the auxin distribution to emerge, confirming the somewhat counterintuitive notion that the VM, although presenting stable low auxin levels, is nevertheless presenting a rich auxin flux pattern. The *in silico* results were built upon the assumption that transporters' intensity and localization in the replum and valve did not alter, which was experimentally verified for PIN3. PIN3 is expressed in the valve and is the only PIN transporter found to be expressed in the replum tissue during fruit development. However, we cannot ascertain that other transporters (besides PIN3) might have been altered due to the chemical ablation, underlying the observed auxin alterations. Nevertheless, even if other transporter patterns did change, this would not negate the flux-passage model *per se* as a likely cause, for such a (non-observed and hypothetical) response would likely be the changes in the auxin patterning or fluxes themselves, and such changes after ablation are only predicted to be triggered within the flux-passage model. We acknowledge that the development of additional crosses between the IND>GR>BARNASE-BARSTAR system and GFP-tagged transporters would be beneficial, since following these inducible lines on a fine-grained timescale would determine exactly if and how changes in auxin and auxin fluxes might unleash transporter modifications, in their turn further affecting the auxin levels and fluxes. Taken together, these results already demonstrate that the flux-passage process is most likely the predominant process, while the flux-barrier process plays a minor role. Such an inverted relationship between low concentrations and high fluxes may be counterintuitive, and can be easily overlooked as a plausible mechanism for patterning.

To our knowledge, the only other instance in which similar correlations have been drawn between low concentrations and high fluxes are the canalization models to describe vein formation (Mitchison, 1980a, Mitchison, 1980b, Mitchison, 1981, Sachs, 1975, Sachs, 1981, Sachs, 1991a, Sachs, 1991b, Feugier et al., 2005). In those models, “with-the-flow” assumptions link PIN positioning to fluxes, yielding low concentrations within veins. Although the thinking behind these models has played a huge part in developing plant systems biology as a discipline, the models themselves incorrectly predicted low auxin levels in the veins, in contrast to the experimentally revealed high auxin concentrations (Mattsson et al., 2003). Here, for the *Arabidopsis* fruit, we support a high flux-low concentration scenario also experimentally. Secondly, canalization models are based on heuristic rules regarding PIN auxin feedbacks, in which it is assumed that cells respond to measuring devices that are not yet supported by known molecular processes, as pointed out by Mitchison, 1980a, Mitchison, 1980b, Mitchison, 1981, Feugier et al., 2005 and others (Bennett et al., 2014). In contrast, we built our model using observed and analyzed molecular biological data. Thirdly, even when not considering the mismatch between actual leaf data and canalization predictions, it remains that the resultant fluxes within these models solely form along the vein/minimum, not across it (Feugier et al., 2005). Hence, the directionality of the fluxes in relation to the orientation of the minimum is an important predictor to distinguish between the processes. We demonstrated empirically that relevant perpendicular fluxes underly the low concentration fields through our ablation interferences, driven by a systems understanding of the auxin minimum formation.

Finally, we have shown that to realistically enable the flux-passage process, an exporter-based scenario is far more robust and likely, for which the inclusion of the cell-wall compartment is essential (Kramer, 2004, el Showk et al., 2015); Grieneisen and Scheres (2009). Interestingly, we here find that a flux-passage process is not only more robust over parameter space but displays additional intriguing behavior, such as effectively connecting tissues over larger domains through fluxes while being separated by a concentration minimum. While it is well established that auxin is developmentally instructive through local concentrations within cells, it has been shown that the auxin fluxes themselves can also be informative (Prusinkiewicz et al., 2009, Bennett et al., 2014, Cieslak et al., 2015). If concentrations and fluxes can indeed be perceived independently by cells, then the decoupling of concentration and flux patterns shown here may help explain some of the amazing versatility of responses to the phytohormone auxin.

## Methods

### Plant Material and Growth Conditions

Plants were grown under long-day conditions (16-h light/8-h dark) at 22°C. Reporter lines of *DR5::GFP* (Friml et al., 2003), *PIN3::PIN3:GFP* (Žádníková et al., 2010), *PIN7::PIN7:GFP* (Blilou et al., 2005), *LAX1::LAX1:VENUS* (Robert et al., 2015), *WIND1::GUS* (Iwase et al., 2011), and PM-mCherry (Nelson et al., 2007) were in Col-0 background. Plants were grown in small individual cells in a glasshouse (maintained at approximately 21°C) in *Arabidopsis* soil mixture (ratio of Levington F2 600 L peat:100 L 4 mm grit:196 g Exemptor (chloronicotinyl insecticide)).

### Construct of *IND::GR≫Barnase-Barstar*

A 2.5 kb *IND* promoter was amplified from *Arabidopsis* genomic DNA and cloned into the Gateway donor vector pDONR207, then recombined into the vector pBIN-LR-LhGR2 (Craft et al., 2005) to produce INDp-LhGR2. The *Barnase-Barstar* coding sequence was cloned into the Gateway donor vector pDONR201, then recombined into the vector pOpIn2 (Craft et al., 2005), which contains pOp6 promoter to produce pOp6-Barnase-Barstar. Finally, the INDp-LhGR2 fusion fragment was digested by AscI and inserted into the vector of pOp6-Barnase-Barstar to generate INDp-LhGR2-pOp6-Barnase-Barstar (IND::GR≫Barnase-Barstar). The construct was introduced into *Agrobacterium tumefaciens* strain AGL1 for transformation into Col-0 plants.

### Dexamethasone Treatment

Inflorescence and siliques were dipped in the solutions containing 10 μM DEX (Sigma-Aldrich) and 0.015% Silwet L-77 for 5 s and treated every 2 days. Relatively mild doses were used to prevent that the ensuing cell death would also cause mechanical separation and disintegration of the fruit tissue, which could be observed at higher doses. At the treatment intensity used, it took several days to a week to obtain complete cell death of the VM. Hence, images of stage 16 fruits (floral organs withering and falling from the fruits) were obtained at 3 days after the first treatment and images of stage 17b fruits (fully elongated and expanded fruits) were taken at 9 days after the first treatment.

### Confocal Microscopy

Fluorescent images were taken on a Zeiss LSM 780 confocal microscope. GFP and chloroplast autofluorescence were excited by 488-nm excitation and mCherry was excited by 514-nm excitation. GFP emission spectra were collected between 499 and 526 nm, autofluorescence was collected between 626 and 695 nm and mCherry was collected between 561 and 602 nm. The images were processed using ImageJ (Schneider et al., 2012).

### GUS Assay

Fruits at stage 17b were collected in Eppendorf tubes containing X-Gluc solution (1 mg/ml X-Gluc (5-bromo-4-chloro-3-indolyl glucuronide; Melford) dissolved in DMSO, 100 mM sodium phosphate buffer, 10 mM EDTA, 0.5 mM K_3_Fe(CN)_6_, 3 mM K_4_Fe(CN)6, 0.1% Triton X-100) and vacuum infiltrated for 30 s, and then incubated at 37°C in the dark for 16 h. The fruits were then treated in 70% ethanol to destain for 2–3 days before taking images on a Leica M205 FA stereo microscope.

### Scanning Electron Microscopy

Fruits were fixed in FAA (50% ethanol, 5% glacial acetic, and 3.7% formaldehyde) for 4 h at room temperature and overnight at 4°C. Samples were then dehydrated through an ethanol series (30 min each in 50%, 60%, 70%, 80%, 90%, 95%, 100%, 100%, and dry ethanol). After critical point drying, samples were coated with gold and examined using a Zeiss Supra 55VP field emission scanning electron microscope.

### Laser Ablation

Tissue ablations were conducted using a Zeiss PLAM MicroBeam microscope, with an inverted 10× objective, a cutting speed of 20 μm/s, and laser power of 65%. These settings were found through trial and error to be the optimum for cutting a single layer of cells while causing minimal damage to the surrounding tissue. Experiments were conducted on individual fruits of the inflorescence meristem, at a stage when the main stem was about 10 cm long. Mature siliques, open flowers, and young buds were removed from the inflorescence, leaving only flowers around stage 15. Prior to ablation, the sepals, petals, and anthers of each flower were removed to reveal the fruit.

For each sample, the inflorescence was placed onto a dry microscope slide, and the rest of the plant was balanced horizontally across the microscope stage. The main stem was adhered to the microscope slide using double-sided sticky tape to hold the gynoecium in the correct orientation. This technique permitted the positioning of the replum roughly perpendicular to the microscope objective. If necessary, an additional microscope slide was placed on top of the gynoecium to hold it in place. Samples were visualized using bright-field illumination. The Zeiss PALM software was used to draw target cutting lines in the location of the VM, which guided the laser path during ablation. Tissue damage was immediately and clearly visualized in the region of ablation as the cells broke open. Samples were imaged (with bright-field illumination) immediately after ablation to confirm the location of the cut site before the plants were returned to the glasshouse to continue growing.

Fruits were removed for imaging at 5 or 6 days after cutting. Double-sided sticky tape was used to mount fruits onto a microscope slide with a drop of 0.1% Silwet L-77 solution (to facilitate imaging through the waxy coating) and topped with a coverslip. Samples were imaged using bright-field illumination on a Leica DM6000 upright light microscope or a Leica SP5 laser confocal scanning microscope, using a 20× immersion objective.

### Video

The confocal Z stack was converted to individual .png files for each slice using ImageJ (Schneider et al., 2012). The converted stack was opened using the 3D visualization software VolViewer (http://cmpdartsvr3.cmp.uea.ac.uk/wiki/BanghamLab/index.php/VolViewer, Lee et al., 2006), and a transfer function was applied to optimize levels. This tool was used to create a series of images, which were saved and then animated using virtual dub (http://virtualdub.sourceforge.net/) before being saved as an .avi file.

## Funding

This work was supported by the Institute Strategic Programme grants (BB/J004588/1 and BB/P013511/1) from the Biotechnology and Biological Sciences Research Council (BBSRC) to the John Innes Centre and by grant BB/M018164/1 to L.Ø. from the FACCE ERA-NET+ and BBSRC.

## Author Contributions

X.-R.L., S.F., V.A.G., A.F.M.M., and L.Ø. planned experiments; X.-R.L. and S.F. performed experiments; V.A.G. and A.F.M.M. planned simulations; R.M.A.V. and A.F.M.M. performed simulations; R.M.A.V., V.A.G., and A.F.M.M. developed the code for simulations; S.F. performed ablation experiments; X.-R.L. developed lines for induction of tissue-specific cell death and performed the experiment; X.-R.L., S.F., and L.Ø. generated microscopy images; X.-R.L., V.A.G., L.Ø., and A.F.M.M. interpreted tissue polarity from data; V.A.G., L.Ø., and A.F.M.M. interpreted all results and wrote the paper; X.-R.L., R.M.A.V., and S.F. edited paper, A.F.M.M., L.Ø., and V.A.G. conceived the research.

## Data and Software Availability

All simulations were performed using computer code written in C developed in-house. A remote repository (Git repository) has been used for the code as well as for the scripts that generated all simulation outputs (graphs and images) that are presented and discussed in the main text and figures and supplemental figures. Details of the computational model are given in the Supplemental Information. Access to the repository is available through Bitbucket after acceptance (https://bitbucket.org/mareeslab/FruitMin).

## Supplemental Information

Document S1. Supplemental Experimental Procedures, Supplemental Figures 1–9, and Supplemental Tables 1 and 2

## References

[bib1] Abley, K., Barbier de Reuille, P., Strutt, D., Bangham, A., Prusinkiewicz, P., Maree, A.F.M., Grieneisen, V.A., Coen, E., 2013. An intracellular partitioning-based framework for tissue cell polarity in plants and animals. Development 140, 2061-2074. 10.1242/dev.062984 23633507

[bib2] Adamowski, M., Friml, J., 2015. PIN-dependent auxin transport: action, regulation, and evolution. Plant Cell 27, 20-32. 10.1105/tpc.114.134874 PMC4330589 25604445

[bib3] Band, L.R., Wells, D.M., Fozard, J.A., Ghetiu, T., French, A.P., Pound, M.P., Wilson, M.H., Yu, L., Li, W., Hijazi, H.I., et al.., 2014. Systems analysis of auxin transport in the Arabidopsis root apex. Plant Cell 26, 862-875. 10.1105/tpc.113.119495 PMC4001398 24632533

[bib4] Bandyopadhyay, A., Blakeslee, J.J., Lee, O.R., Mravec, J., Sauer, M., Titapiwatanakun, B., Makam, S.N., Bouchard, R., Geisler, M., Martinoia, E., et al.., 2007. Interactions of PIN and PGP auxin transport mechanisms. Biochem. Soc. Trans. 35, 137-141. 10.1042/BST0350137 17233620

[bib5] Barbier de Reuille, P., Bohn-Courseau, I., Ljung, K., Morin, H., Carraro, N., Godin, C., Traas, J., 2006. Computer simulations reveal properties of the cell-cell signaling network at the shoot apex in Arabidopsis. Proc. Natl. Acad. Sci. U S A 103, 1627-1632. 10.1073/pnas.0510130103 PMC1360567 16432202

[bib6] Beals, T.P., Goldberg, R.B., 1997. A novel cell ablation strategy blocks tobacco anther dehiscence. Plant Cell 9, 1527-1545. 10.1105/tpc.9.9.1527 PMC157031 9338959

[bib7] Benjamins, R., Quint, A., Weijers, D., Hooykaas, P., Offringa, R., 2001. The PINOID protein kinase regulates organ development in Arabidopsis by enhancing polar auxin transport. Development 128, 4057-4067. 10.1242/dev.128.20.4057 11641228

[bib8] Benkova, E., Michniewicz, M., Sauer, M., Teichmann, T., Seifertova, D., Jurgens, G., Friml, J., 2003. Local, efflux-dependent auxin gradients as a common module for plant organ formation. Cell 115, 591-602. 10.1016/s0092-8674(03)00924-3 14651850

[bib9] Bennett, T., Hines, G., Leyser, O., 2014. Canalization: what the flux? Trends Genet. 30, 41-48. 10.1016/j.tig.2013.11.001 24296041

[bib10] Bilsborough, G.D., Runions, A., Barkoulas, M., Jenkins, H.W., Hasson, A., Galinha, C., Laufs, P., Hay, A., Prusinkiewicz, P., Tsiantis, M., 2011. Model for the regulation of Arabidopsis thaliana leaf margin development. Proc. Natl. Acad. Sci. U S A. 108, 3424-3429. 10.1073/pnas.1015162108 PMC3044365 21300866

[bib11] Blilou, I., Xu, J., Wildwater, M., Willemsen, V., Paponov, I., Friml, J., Heidstra, R., Aida, M., Palme, K., Scheres, B., 2005. The PIN auxin efflux facilitator network controls growth and patterning in Arabidopsis roots. Nature 433, 39-44. 10.1038/nature03184 15635403

[bib12] Brunoud, G., Wells, D.M., Oliva, M., Larrieu, A., Mirabet, V., Burrow, A.H., Beeckman, T., Kepinski, S., Traas, J., Bennett, M.J., et al.., 2012. A novel sensor to map auxin response and distribution at high spatio-temporal resolution. Nature 482, 103-106. 10.1038/nature10791 22246322

[bib13] Caggiano, M.P., Yu, X., Bhatia, N., Larsson, A., Ram, H., Ohno, C.K., Sappl, P., Meyerowitz, E.M., Jonsson, H., Heisler, M.G., 2017. Cell type boundaries organize plant development. Elife 6, e27421. 10.7554/eLife.27421 PMC5617630 28895530

[bib14] Cheng, Y., Dai, X., Zhao, Y., 2006. Auxin biosynthesis by the YUCCA flavin monooxygenases controls the formation of floral organs and vascular tissues in Arabidopsis. Genes Dev. 20, 1790-1799. 10.1101/gad.1415106 PMC1522075 16818609

[bib15] Cieslak, M., Runions, A., Prusinkiewicz, P., 2015. Auxin-driven patterning with unidirectional fluxes. J. Exp. Bot. 66, 5083-5102. 10.1093/jxb/erv262 PMC4513925 26116915

[bib16] Craft, J., Samalova, M., Baroux, C., Townley, H., Martinez, A., Jepson, I., Tsiantis, M., Moore, I., 2005. New pOp/LhG4 vectors for stringent glucocorticoid-dependent transgene expression in Arabidopsis. Plant J. 41, 899-918. 10.1111/j.1365-313X.2005.02342.x 15743453

[bib17] Dubrovsky, J.G., Sauer, M., Napsucialy-Mendivil, S., Ivanchenko, M.G., Friml, J., Shishkova, S., Celenza, J., Benkova, E., 2008. Auxin acts as a local morphogenetic trigger to specify lateral root founder cells. Proc. Natl. Acad. Sci. U S A. 105, 8790-8794. 10.1073/pnas.0712307105 PMC2438385 18559858

[bib18] Eklund, D.M., Staldal, V., Valsecchi, I., Cierlik, I., Eriksson, C., Hiratsu, K., Ohme-Takagi, M., Sundstrom, J.F., Thelander, M., Ezcurra, I., et al.., 2010. The Arabidopsis thaliana STYLISH1 protein acts as a transcriptional activator regulating auxin biosynthesis. Plant Cell 22, 349-363. 10.1105/tpc.108.064816 PMC2845406 20154152

[bib19] Feugier, F.G., Mochizuki, A., Iwasa, Y., 2005. Self-organization of the vascular system in plant leaves: inter-dependent dynamics of auxin flux and carrier proteins. J. Theor. Biol. 236, 366-375. 10.1016/j.jtbi.2005.03.017 15899502

[bib20] Friml, J., Vieten, A., Sauer, M., Weijers, D., Schwarz, H., Hamann, T., Offringa, R., Jurgens, G., 2003. Efflux-dependent auxin gradients establish the apical-basal axis of Arabidopsis. Nature 426, 147-153. 10.1038/nature02085 14614497

[bib21] Friml, J., Yang, X., Michniewicz, M., Weijers, D., Quint, A., Tietz, O., Benjamins, R., Ouwerkerk, P.B., Ljung, K., Sandberg, G., et al.., 2004. A PINOID-dependent binary switch in apical-basal PIN polar targeting directs auxin efflux. Science 306, 862-865. 10.1126/science.1100618 15514156

[bib22] Geisler, M., Aryal, B., di Donato, M., Hao, P., 2017. A critical view on ABC transporters and their interacting partners in auxin transport. Plant Cell Physiol. 58, 1601-1614. 10.1093/pcp/pcx104 29016918

[bib23] van Gelderen, K., van Rongen, M., Liu, A., Otten, A., Offringa, R., 2016. An INDEHISCENT-controlled auxin response specifies the separation layer in early Arabidopsis fruit. Mol. Plant 9, 857-869. 10.1016/j.molp.2016.03.005 26995296

[bib24] Grieneisen, V.A., Scheres, B., 2009. Back to the future: evolution of computational models in plant morphogenesis. Curr. Opin. Plant Biol. 12, 606-614. 10.1016/j.pbi.2009.07.008 19709922

[bib25] Grieneisen, V.A., Xu, J., Maree, A.F.M., Hogeweg, P., Scheres, B., 2007. Auxin transport is sufficient to generate a maximum and gradient guiding root growth. Nature 449, 1008-1013. 10.1038/nature06215 17960234

[bib26] Grieneisen, V.A., Scheres, B., Hogeweg, P., Maree, A.F.M., 2012. Morphogengineering roots: comparing mechanisms of morphogen gradient formation. BMC Syst. Biol. 6, 37. 10.1186/1752-0509-6-37 PMC3681314 22583698

[bib27] Grieneisen, V.A., Maree, A.F.M., Ostergaard, L., 2013. Juicy stories on female reproductive tissue development: coordinating the hormone flows. J. Integr. Plant Biol. 55, 847-863. 10.1111/jipb.12092 23869979

[bib28] Han, X., Hyun, T.K., Zhang, M., Kumar, R., Koh, E.J., Kang, B.H., Lucas, W.J., Kim, J.Y., 2014. Auxin-callose-mediated plasmodesmal gating is essential for tropic auxin gradient formation and signaling. Dev. Cell 28, 132-146. 10.1016/j.devcel.2013.12.008 24480642

[bib29] Heisler, M.G., Hamant, O., Krupinski, P., Uyttewaal, M., Ohno, C., Jonsson, H., Traas, J., Meyerowitz, E.M., 2010. Alignment between PIN1 polarity and microtubule orientation in the shoot apical meristem reveals a tight coupling between morphogenesis and auxin transport. PLoS Biol. 8, e1000516. 10.1371/journal.pbio.1000516 PMC2957402 20976043

[bib30] Iwase, A., Mitsuda, N., Koyama, T., Hiratsu, K., Kojima, M., Arai, T., Inoue, Y., Seki, M., Sakakibara, H., Sugimoto, K., et al.., 2011. The AP2/ERF transcription factor WIND1 controls cell dedifferentiation in Arabidopsis. Curr. Biol. 21, 508-514. 10.1016/j.cub.2011.02.020 21396822

[bib31] Jacob, F., Philip, F., 1995. The Statue within: An Autobiography. Cold Spring Harbor Press, Cold Spring Harbor, NY.

[bib32] Kramer, E.M., 2004. PIN and AUX/LAX proteins: their role in auxin accumulation. Trends Plant Sci. 9, 578-582. 10.1016/j.tplants.2004.10.010 15564124

[bib33] Kuusk, S., Sohlberg, J.J., Long, J.A., Fridborg, I., Sundberg, E., 2002. STY1 and STY2 promote the formation of apical tissues during Arabidopsis gynoecium development. Development 129, 4707-4717. 10.1242/dev.129.20.4707 12361963

[bib34] Laskowski, M., Grieneisen, V.A., Hofhuis, H., ten Hove, C.A., Hogeweg, P., Maree, A.F.M., Scheres, B., 2008. Root system architecture from coupling cell shape to auxin transport. PLoS Biol. 6, e307. 10.1371/journal.pbio.0060307 PMC2602721 19090618

[bib35] Lee, K., Avondo, J., Morrison, H., Blot, L., Stark, M., Sharpe, J., Bangham, A., Coen, E., 2006. Visualizing plant development and gene expression in three dimensions using optical projection tomography. Plant Cell 18, 2145-2156. 10.1105/tpc.106.043042 PMC1560903 16905654

[bib36] Liljegren, S.J., Roeder, A.H.K., Kempin, S.A., Gremski, K., Ostergaard, L., Guimil, S., Reyes, D.K., Yanofsky, M.F., 2004. Control of fruit patterning in Arabidopsis by INDEHISCENT. Cell 116, 843-853. 10.1016/s0092-8674(04)00217-x 15035986

[bib37] Di Mambro, R., De Ruvo, M., Pacifici, E., Salvi, E., Sozzani, R., Benfey, P.N., Busch, W., Novak, O., Ljung, K., Di Paola, L., et al.., 2017. Auxin minimum triggers the developmental switch from cell division to cell differentiation in the Arabidopsis root. Proc. Natl. Acad. Sci. U S A. 114, E7641-E7649. 10.1073/pnas.1705833114 PMC5594665 28831001

[bib38] Marchant, A., Bhalerao, R., Casimiro, I., Eklof, J., Casero, P.J., Bennett, M., Sandberg, G., 2002. AUX1 promotes lateral root formation by facilitating indole-3-acetic acid distribution between sink and source tissues in the Arabidopsis seedling. Plant Cell 14, 589-597. 10.1105/tpc.010354 PMC150581 11910006

[bib39] Mattsson, J., Ckurshumova, W., Berleth, T., 2003. Auxin signaling in Arabidopsis leaf vascular development. Plant Physiol. 131, 1327-1339. 10.1104/pp.013623 PMC166892 12644682

[bib40] Mitchison, G.J., 1980a. The dynamics of auxin transport. Proc. R. Soc. Lond. B 209, 489-511.

[bib41] Mitchison, G.J., 1980b. A model for vein formation in higher plants. Proc. R. Soc. Lond. B 207, 79-109.

[bib42] Mitchison, G.J., 1981. The polar transport of auxin and vein patterns in plants. Philos. Trans. R. Soc. Lond. B 295, 461-471.

[bib43] Nelson, B.K., Cai, X., Nebenfuhr, A., 2007. A multicolored set of in vivo organelle markers for co-localization studies in Arabidopsis and other plants. Plant J. 51, 1126-1136. 10.1111/j.1365-313X.2007.03212.x 17666025

[bib44] Park, J., Lee, Y., Martinoia, E., Geisler, M., 2017. Plant hormone transporters: what we know and what we would like to know. BMC Biol. 15, 93. 10.1186/s12915-017-0443-x PMC5655956 29070024

[bib45] Payne, R.J.H., Grierson, C.S., 2009. A theoretical model for ROP localisation by auxin in Arabidopsis root hair cells. PLoS One. 4, e8337. 10.1371/journal.pone.0008337 PMC2791196 20016781

[bib46] Petersen, M., Sander, L., Child, R., van Onckelen, H., Ulvskov, P., Borkhardt, B., 1996. Isolation and characterisation of a pod dehiscence zone-specific polygalacturonase from Brassica napus. Plant Mol. Biol. 31, 517-527. 10.1007/BF00042225 8790285

[bib47] Prusinkiewicz, P., Crawford, S., Smith, R.S., Ljung, K., Bennett, T., Ongaro, V., Leyser, O., 2009. Control of bud activation by an auxin transport switch. Proc. Natl. Acad. Sci. U S A 106, 17431-17436. 10.1073/pnas.0906696106 PMC2751654 19805140

[bib48] Raspopovic, J., Marcon, L., Russo, L., Sharpe, J., 2014. Modeling digits. Digit patterning is controlled by a Bmp-Sox9-Wnt Turing network modulated by morphogen gradients. Science 345, 566-570. 10.1126/science.1252960 25082703

[bib49] Reinhardt, D., Pesce, E.R., Stieger, P., Mandel, T., Baltensperger, K., Bennett, M., Traas, J., Friml, J., Kuhlemeier, C., 2003. Regulation of phyllotaxis by polar auxin transport. Nature 426, 255-260. 10.1038/nature02081 14628043

[bib50] Robert, H.S., Grunewald, W., Sauer, M., Cannoot, B., Soriano, M., Swarup, R., Weijers, D., Bennett, M., Boutilier, K., Friml, J., 2015. Plant embryogenesis requires AUX/LAX-mediated auxin influx. Development 142, 702-711. 10.1242/dev.115832 25617434

[bib51] Roeder, A.H.K., Yanofsky, M.F., 2006. Fruit development in Arabidopsis. Arabidopsis Book 4, e0075. 10.1199/tab.0075 PMC3243326 22303227

[bib52] Sabatini, S., Beis, D., Wolkenfelt, H., Murfett, J., Guilfoyle, T., Malamy, J., Benfey, P., Leyser, O., Bechtold, N., Weisbeek, P., et al.., 1999. An auxin-dependent distal organizer of pattern and polarity in the Arabidopsis root. Cell 99, 463-472. 10.1016/s0092-8674(00)81535-4 10589675

[bib53] Sachs, T., 1975. The control of the differentiation of vascular networks. Ann. Bot. 39, 197-204.

[bib54] Sachs, T., 1981. The control of the patterned differentiation of vascular tissues. Adv. Bot. Res. 9, 151-262.

[bib55] Sachs, T., 1991a. Cell polarity and tissue patterning in plants. Development 113, 83-93.

[bib56] Sachs, T., 1991b. Pattern Formation in Plant Tissues. Cambridge University Press, Cambridge.

[bib57] Scarpella, E., Marcos, D., Friml, J., Berleth, T., 2006. Control of leaf vascular patterning by polar auxin transport. Genes Dev. 20, 1015-1027. 10.1101/gad.1402406 PMC1472298 16618807

[bib58] Schneider, C.A., Rasband, W.S., Eliceiri, K.W., 2012. NIH Image to ImageJ: 25 years of image analysis. Nat. Methods 9, 671-675. 10.1038/nmeth.2089 PMC5554542 22930834

[bib59] el Showk, S., Help-Rinta-Rahko, H., Blomster, T., Siligato, R., Maree, A.F.M., Mahonen, A.P., Grieneisen, V.A., 2015. Parsimonious model of vascular patterning links transverse hormone fluxes to lateral root initiation: auxin leads the way, while cytokinin levels out. PLoS Comput. Biol. 11, e1004450. 10.1371/journal.pcbi.1004450 PMC4623515 26505899

[bib60] Smith, R.S., Guyomarc’h, S., Mandel, T., Reinhardt, D., Kuhlemeier, C., Prusinkiewicz, P., 2006. A plausible model of phyllotaxis. Proc. Natl. Acad. Sci. U S A. 103, 1301-1306. 10.1073/pnas.0510457103 PMC1345713 16432192

[bib61] Smyth, D.R., Bowman, J.L., Meyerowitz, E.M., 1990. Early flower development in Arabidopsis. Plant Cell 2, 755-767. 10.1105/tpc.2.8.755 PMC159928 2152125

[bib62] Sorefan, K., Girin, T., Liljegren, S.J., Ljung, K., Robles, P., Galvan-Ampudia, C.S., Offringa, R., Friml, J., Yanofsky, M.F., Ostergaard, L., 2009. A regulated auxin minimum is required for seed dispersal in Arabidopsis. Nature 459, 583-586. 10.1038/nature07875 19478783

[bib63] Spence, J., Vercher, Y., Gates, P., Harris, N., 1996. ‘Pod shatter’ in Arabidopsis thaliana, Brassica napus and B. juncea. J. Microsc. 181, 195-203.

[bib64] Stoma, S., Lucas, M., Chopard, J., Schaedel, M., Traas, J., Godin, C., 2008. Flux-based transport enhancement as a plausible unifying mechanism for auxin transport in meristem development. PLoS Comput. Biol. 4, e1000207. 10.1371/journal.pcbi.1000207 PMC2565506 18974825

[bib65] Swarup, R., Peret, B., 2012. AUX/LAX family of auxin influx carriers - an overview. Front. Plant Sci. 3, 225. 10.3389/fpls.2012.00225 PMC3475149 23087694

[bib66] Ullmann, A., 2011. In memoriam: Jacques Monod (1910-1976). Genome Biol. Evol. 3, 1025-1033. 10.1093/gbe/evr024 PMC3264052 21979155

[bib67] Wolpert, L., 2016. Positional information and pattern formation. Curr. Top. Dev. Biol. 117, 597-608. 10.1016/bs.ctdb.2015.11.008 26970003

[bib68] Wu, H., Mori, A., Jiang, X., Wang, Y., Yang, M., 2006. The INDEHISCENT protein regulates unequal cell divisions in Arabidopsis fruit. Planta 224, 971-979. 10.1007/s00425-006-0351-8 16845525

[bib69] Žadnikova, P., Petrašek, J., Marhavý, P., Raz, V., Vandenbussche, F., Ding, Z., Schwarzerova, K., Morita, M.T., Tasaka, M., Hejatko, J., et al.., 2010. Role of PIN-mediated auxin efflux in apical hook development of Arabidopsis thaliana. Development 137, 607-617. 10.1242/dev.041277 20110326

