# Supplementary material for: Systems Biology Approach Pinpoints Minimum Requirements for Auxin Distribution during Fruit Opening
Source: Mol Plant. 2019 Jun 3;12(6):863–78. doi: 10.1016/j.molp.2019.05.003 (PMC6557309; doi:10.1016/j.molp.2019.05.003)
Supplement: Document S1. Supplemental Experimental Procedures, Supplemental Figures 1–9, and Supplemental Tables 1 and 2 [file mmc1.pdf]

Supplemental Information for

Systems biology approach pinpoints minimum requirements for auxin distribution during fruit opening

Xin-Ran Li[a,1], Renske M. A. Vroomans[b,c,1], Samantha Fox[d], Verônica A. Grieneisen[b,e], Lars Østergaard[a,*], Athanasius F. M. Marée[b,e,*]

[a]*Crop Genetics, John Innes Centre, Norwich NR4 7UH, UK.*
[b]*Computational and Systems Biology, John Innes Centre, Norwich NR4 7UH, UK.*
[c]*Centre of Excellence in Computational and Experimental Developmental Biology, Institute of Biotechnology, University of Helsinki, FIN-00014 Helsinki, Finland.*
[d]*Cell and Developmental Biology, John Innes Centre, Norwich NR4 7UH, UK.*
[e]*School of Biosciences, Cardiff University, Cardiff, CF10 3AX, Wales, UK.*

This Supplemental Information includes detailed *Computational Procedures* and *Quantification and Statistical Analysis*, plus *nine figures*, *two tables* and *one movie*.

[*]To whom correspondence should be addressed.
Email addresses: `lars.ostergaard@jic.ac.uk` and `marees@cardiff.ac.uk`
[1]X.-R.L. and R.M.A.V. contributed equally to this work.

Supplemental Information

Detailed Computational Procedures

Simulating the fruit

Auxin dynamics were simulated using a spatially explicit and multicellular layout of an *Arabidopsis* fruit at early stages of development (between 15–17), incorporating subcellular resolution concerning transporter activity in a tissue-specific manner, as well as explicitly modelling the cell wall. Our general modelling strategy was to analyse the steady states distributions given alternative hypotheses on auxin transporter levels and polarity, analysing both auxin profiles and flux patterns. Importantly, given that some features regarding auxin distributions were indistinguishable between different models, but the underlying flux patterns were not, we performed *in silico* ablations and observed the resultant auxin distributions. This process is explained in detail below.

Fruit layout

Our model consists of a 2D sheet of cells, intercalated by the contiguous network of cell wall, representing the outer epidermis of the developing *Arabidopsis* fruit. On this field, we employ wrapped boundary conditions in the horizontal direction. That is, the right-most pixels of the field (at L) are coupled on the right to the left-most pixels of the field, at position 0, such that $L + 1 = 0$. Therefore, the 2D sheet represents a closed cylinder, describing the outer tissue of the fruit. We always perform simulations on the entire external epidermis, although we often only display half of the field (when presenting the same pattern as the other half, due to symmetry reasons). In the y-direction, we have fixed boundary conditions to represent the top and bottom of the fruit. Because of constraints imposed by the duration of the computer simulations, the fruit was simulated at two different sizes, as indicated in Table S1. Cell sizes were conserved between both layouts, representing realistic cell sizes, as measured during stage 17b. The conceptual models, however, were run on a much smaller, simplified tissue layout, while the realistic models were run on the exact, measured tissue layout.

Special assumptions regarding auxin sources and sinks at these positions were introduced. To capture the auxin source at the style, we set the top-most row of cells of the fruit tissue to a fixed concentration a_0 that does not alter during the simulation (see Table S2 for parameter values), while also imposing that these cells only permit efflux of auxin into the fruit tissue. These assumptions ensure that the top-most row of the fruit acts as a perfect source without a sink term. In conjunction, the bottom-most row of cells of the *in silico* fruit have concentrations set to zero, hence acting as a perfect sink.

Within this 2D epidermal representation, the four different tissues were positioned, always in a similar manner. They included the replum, the valve, the lignifying layer and the separation layer. The number of cell files and columns were based on careful examination of fruit layouts, and their values are given in Table S1. The tissues also differ with respect to properties regarding transporters and cellular polarity, i.e., which transporters are expressed, how strongly they are positioned at the plasma membrane, and their orientation along the plasma membrane, as described in the next section.

Cell polarity

The cells were subdivided into eight regions, as depicted in Figure S1. Transporter levels on the membranes within each region could be adjusted according to experimental observations of cell polarity and transporter densities. The regions were defined according to their relative position of the cell within the tissue context. "Upper" and "Lower" sides are equal for all cells of the fruit; "Inner" and "Outer" refer to the facet of the cell facing the closest central replum axis of the fruit. Due to these 4 main directions, combinations can be derived. "Upper-Inner" and "Upper-Outer" correspond to the two halves of the apical side of the cell, "Lower-Inner" and "Lower-Outer" to the two halves of the basal side. Likewise, "Inner-Lower" and "Inner-Upper" describe the top-most and lower-most half facet of the cell facing the nearest middle of the replum. These detailed position-dependent transporter levels are then defined for each tissue type, based upon either published data (for the basic model and its derivatives) or upon careful novel experimental observations (for the realistic model). This generates for each transporter in the model and for each given tissue type eight possible entries to describe the permeability strength of the transporter at the different locations along the cell's plasma membrane. As we have 3 transporters, we therefore have three 8×4 matrixes for each simulation condition. The elements of the matrix for each transporter are scaled from 0 (no transporter of that type at that position of the membrane) to 1 (maximum expression).

We have explored in this paper several variations of transporter patterning, both "toy-models", that represented extreme assumptions, and more nuanced versions, in which experimental observations motivated the choices of transporter levels and positions for different stages of the fruit development. The full set of matrixes used are given in Table 1 and 2.

Auxin dynamics

Changes in auxin concentration over time result from the contribution of transport terms (diffusion and directed or passive permeability) and reaction terms (biosynthesis and breakdown):

$$\frac{dC}{dt} = -\nabla \cdot \vec{J} + b - \delta_{IAA} C \,. \tag{1}$$

The time change in auxin concentration is given by the transport term, due to auxin fluxes $\vec{J}$, and to the reaction terms b, representing biosynthesis, and δ_{IAA}, representing degradation. Note that we are able to distinguish diffusion from permeability, because we explicitly treat the cell as having an interior cytoplasm as well as an external apoplastic region in which auxin can diffuse. Movement across the membrane is included through the permeability terms.

Transport terms

Auxin diffuses within the cell wall and cytoplasm. It crosses the membrane either through passive permeability, for which influx is greater than efflux (because of chemiosmotic considerations), or due to directed influx, mediated by LAX1, or polar efflux, due to PINs. When transport across the membrane occurs, auxin moves between the cytoplasm and the cell wall.

Diffusion within cells and cell walls follows Fick's law, while transport across cell membranes includes both passive and cell-type specific carrier-mediated influx and efflux, and is considered linearly dependent on the concentration. Thus, auxin flux across each grid point was computed as:

$$J = \begin{cases} -D \cdot \nabla c & \text{within cell or cell wall}\,, \\ -P_{out}\hat{n} \cdot C_{in} + P_{in}\hat{n} \cdot C_{out} & \text{accross the membrane}\,, \end{cases} \tag{2}$$

where fluxes due to diffusion occur in the cell wall and cytoplasm with diffusivity D (upper part of equation). The flux across the cell membrane is determined by the resultant auxin permeability across a cell membrane due to the different contributions of transporters at that location. This results in an effective outward (P_{out}) and inward (P_{in}) permeability rate, with n representing the inward directed unit vector, perpendicular to the membrane; c_{in} the auxin concentration in the cytosol at the grid point bordering the cell membrane and c_{out} the auxin concentration in the cell wall grid point immediately adjacent to the cell membrane.

The influx term P_{in} is given by the sum of the passive influx permeability (P_{IAAH}), and the permeability due to LAX1 expression (P_{LAX1}), itself given by the maximum strength of LAX1 transport times the expression of LAX at that region, i.e. $P_{LAX1} = M_{LAX1} \cdot E_{LAX1}$, hence:

$$P_{in} = P_{IAAH} + P_{LAX1} \,. \tag{3}$$

Similarly, the efflux term P_{out} is given by the sum of the passive efflux permeability (P_{IAA^-}), and the efflux permeabilities due to the PINs (P_{PIN3}, P_{PIN7}):

$$P_{out} = P_{IAA^-} + P_{PIN3} + P_{PIN7} \,, \tag{4}$$

with, again, $P_{PIN3} = M_{PIN3} \cdot E_{PIN3}$ and $P_{PIN7} = M_{PIN7} \cdot E_{PIN7}$. All default parameters are given in Table S2, and specified differently if changed.

Reaction Terms

Auxin reaction terms (shown in Equation 1) include a basal biosynthesis rate b and a basal decay δ_{IAA} (i.e. auxin half-life, considering also basic auxin catabolism). These terms take place inside all cells (not in the cell walls), following the framework and realistic values as measured and used in Di Mambro et al. (2017).

To illustrate the feasibility of an alternative mechanism in which the patterning arises through spatial variations in production and decay, as inspired by traditional reaction-diffusion models, we also explored the possible role of localised production and decay to the pattern formation of a minimum. We did so by considering all replum and valve tissue acting as an auxin source, while the VM was bestowed with an increased auxin breakdown. The default parameters b and δ_{IAA} (as given in Table S2) were in this specific case varied as described in the caption of Figure S3.

Numerical framework

The partial differential equation describing auxin dynamics (Equation 1) was solved numerically by means of discretising it on the defined lattice, and concurrently solving for the diffusion, permeability, and production and decay of auxin, using the Alternating Direction Implicit (ADI) method, as implemented in our previous works (Cruz-Ramírez et al., 2012; Di Mambro et al., 2017). A space step Δx corresponding to 1 µm and a time step Δt corresponding to 0.1 s were chosen. Boundary conditions are as described above, roughly, with an auxin source at the top most row, and sink at the bottom most row. For initial condition for simulations, auxin concentration were set to 0 within the whole tissue. As soon as auxin starts to flow into the fruit from the apical cells, a pattern in auxin distribution emerges. Auxin distributions and heat maps were obtained letting each simulation reach steady state, which we ensured had happened when only negligible changes occurred (below $10^{-5}\%$ for each numerically calculated position) in local auxin concentrations.

Quantification and Statistical Analysis

Tissue layout

The tissue layout that was used in the realistic simulations has been based upon our own measurements on cell and tissue dimensions using microscopy images at stage 17b. The cell and tissue sizes as are given in Table S1 were obtained by analysing three images at stage 17b and measuring multiple individual cells per tissue.

Quantification of PIN and LAX level and localisation

Intensity and localisation of fluorescently tagged PIN3, PIN7 and LAX1 proteins for each tissue type and each facet (as defined in Figure S1) were evaluated visually by four of the authors, using a minimum of three confocal images for each transporter and at each developmental stage. Cells were assessed using values from 0 to 1, with 0 = undetectable and 1 = maximum intensity. We opted for semi-quantitative, visual inspection using 3-4 images for each transporter, as automatic quantification renders itself extremely difficult due to the 3D nature of the silique's surface. Visually estimated expression values and localisation of all transporters were then discussed and consensus values found. These final semi-quantitative estimates were used to instruct the "realistic PIN and LAX1" simulations of our model. No *a posteriori* modifications were made to any of the simulation parameter value entries.

Averaging flux-field properties over the tissue for flux analysis

Auxin moves over the simulated tissue following the resultant auxin flux $\vec{J}(x,y) = J_x\hat{\imath} + J_y\hat{\jmath}$. To analyse the differences in flux behaviour between the different models within certain regions of the fruit tissue, we found it useful to compare and contrast **(i)** the average horizontal flux components (J_x) over the valve margin (VM); **(ii)** the average vertical flux components (J_y) within the VM; as well as **(iii)** the averaged norm of the fluxes ($\|\vec{J}\|$) that take place within the VM. The average transversal flux through the VM is calculated as follows:

$$\overline{J_x} = \frac{\iint_S J_x dxdy}{\text{area of S}}, \tag{5}$$

where S is the entire surface of the VM, as depicted in Figure 1. The average flux parallel to the VM is calculated similarly, i.e., $\overline{J_y} = \left(\iint_S J_y dxdy\right)/(\text{area of S})$. For the average total flux, we calculate

$$\overline{\|\vec{J}\|} = \frac{\iint_S \|\vec{J}\| dxdy}{\text{area of S}}. \tag{6}$$

Supporting References

Cruz-Ramírez, A., Díaz-Triviño, S., Blilou, I., Grieneisen, V.A., Sozzani, R., Zamioudis, C., Miskolczi, P., Nieuwland, J., Benjamins, R., Dhonukshe, P., Caballero-Pérez, J., Horvath, B., Long, Y., Mähönen, A.P., Zhang, H., Xu, J., Murray, J.A., Benfey, P.N., Bako, L., Marée, A.F.M., Scheres, B., 2012. A bistable circuit involving SCARECROW-RETINOBLASTOMA integrates cues to inform asymmetric stem cell division. Cell 150, 1002–1015. URL: http://dx.doi.org/10.1016/j.cell.2012.07.017, doi:10.1016/j.cell.2012.07.017.

Di Mambro, R., De Ruvo, M., Pacifici, E., Salvi, E., Sozzani, R., Benfey, P.N., Busch, W., Novak, O., Ljung, K., Di Paola, L., Marée, A.F.M., Costantino, P., Grieneisen, V.A., Sabatini, S., 2017. Auxin minimum triggers the developmental switch from cell division to cell differentiation in the *Arabidopsis* root. Proc. Natl. Acad. Sci. U.S.A. 114, E7641–E7649. URL: http://dx.doi.org/10.1073/pnas.1705833114, doi:10.1073/pnas.1705833114.

Supplemental Figures, Tables and Movie

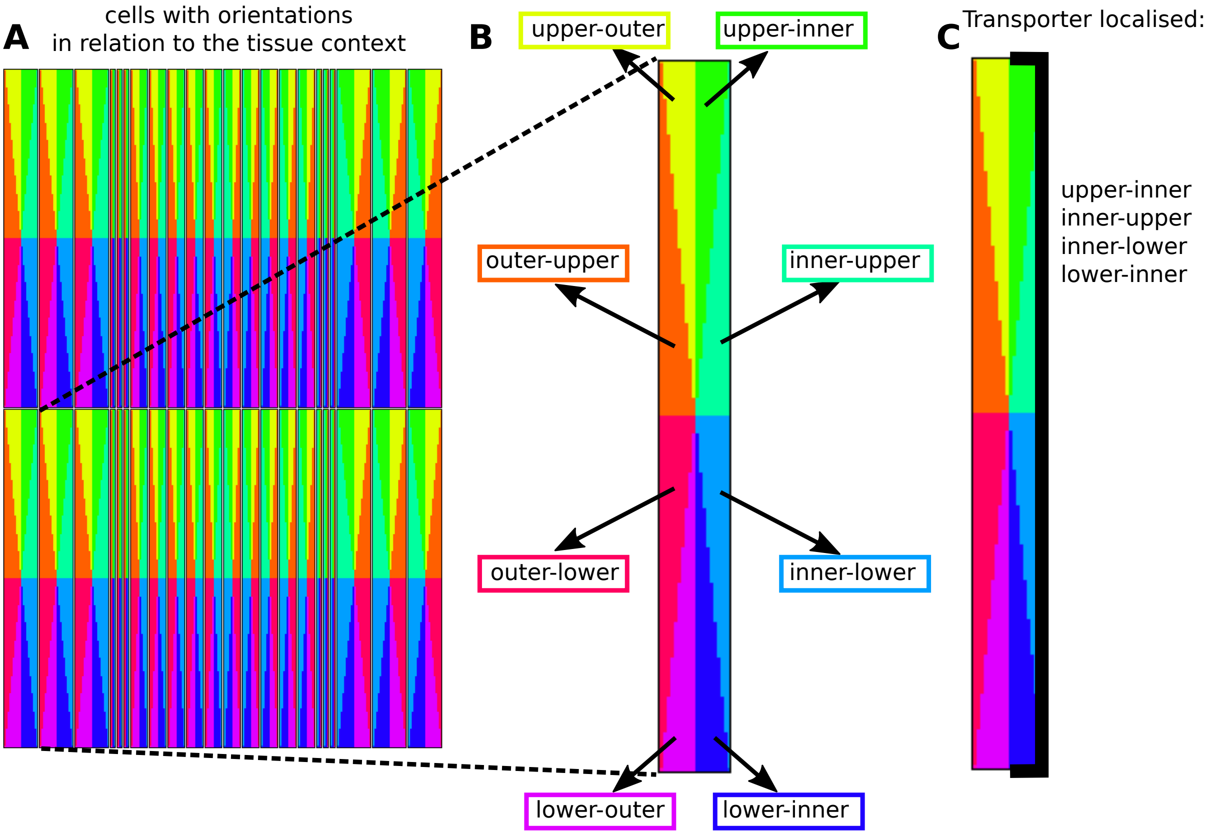

Figure S1: **Model implementation details. Related to Figure 1.** **(A)** Polarity within tissue is defined relative to the closest mid-replum axis, as shown. From these reference axes (two per fruit), **(B)** polarity is assigned to each cell, using the 8 indicated directions highlighted. These reference axes also used to define which fluxes should be considered to move either "inwards" or "outwards" along the horizontal axis (while along the vertical axis, 'up' and 'down' have the same meaning throughout the whole tissue). **(C)** Example of polar transporter localisation, with upper-inner, inner-upper, inner-lower and lower-inner facets occupied.

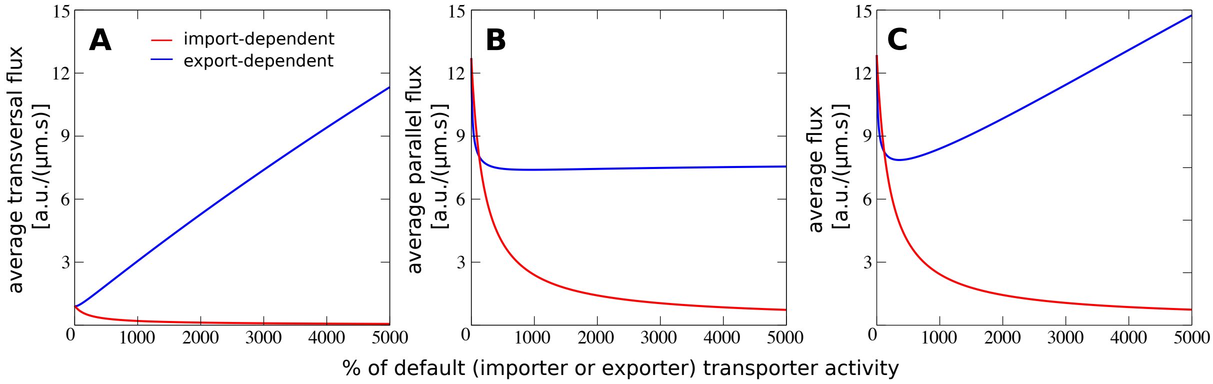

Figure S2: **Decomposed fluxes over the VM, to compare and contrast import-based and export-based models for different degrees of transporter activity levels. Related to Figure 2. (A)** Strengthening apolar exporters in the VM (blue line, export-dependent), which deepens the minimum (Figure 2), also increases the fluxes that cross the VM region; whereas increasing the augmented influx activity in all tissues except the VM (red line), strongly decreases these transversal flows. The average transversal flux occurring within the VM regions is plotted along the y-axis. **(B)** Similarly, the effect of changing those transporter rates on the total fluxes occurring along the VM (i.e., parallel to the VM, along the vertical direction), shows that for the import-dependent case (red line) strong transport strongly decreases those parallel fluxes, while for the export-dependent case only a limited decrease can be observed. **(C)** When combining both flux components (perpendicular and parallel to the VM) in the form of the average flux magnitudes within the VM region, it can again be observed that the fluxes change very differently in the export- (blue), and import- (red) dependent case, for most parameter values presenting a much higher average flux in the export-dependent model. Calculation of the values of the average perpendicular and parallel fluxes over and within the VM as well the flux intensity vector lengths averaged over the VM are described in the the Supplemental Information.

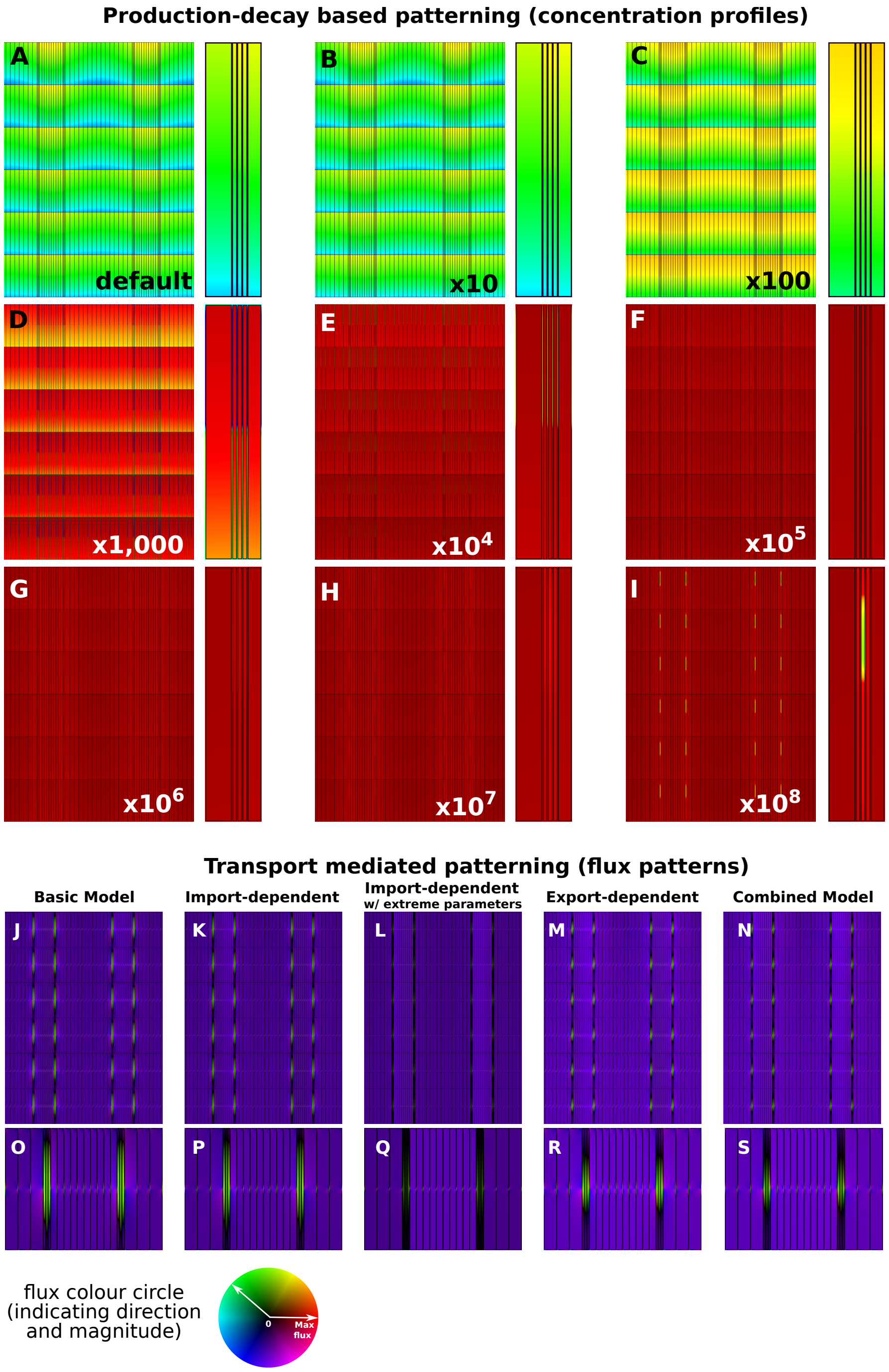

Figure S3: **Production-decay model and tissue fluxes of transport-based models. Related to Figure 2. (A–I)** Model of fruit in which patterning relies on localised production and degradation only (see Table 1), with insets to right of (A-I) magnifying a portion of the left VM, to show for (A–H) that no auxin minimum is being formed, while only in (I) a 'minimum' is found, albeit very weak compared to the minima formed through any of the transport-based mechanisms (Figure 2). **(A)** Lack of patterning when using the default production and degradation values as given in Table S2. **(B–I)** Fold increases in both production and degradation with respect to default values: **(B)** 10-fold increase; **(C)** 100-fold increase; **(D)** 1,000-fold increase; **(E)** 10^4-fold increase; **(F)** 10^5-fold increase; **(G)** 10^6-fold increase; **(H)** 10^7-fold increase; and **(I)** 10^8-fold increase. At a 10^7-fold increase a faint pattern of lower values at the VM appears; only at a 10^8-fold increase a clear minimum is established. **(J–S)** Flux distributions over the tissue are shown, through a colour circle that represents the direction (colours around the circle indicate the direction of the flux vector), as well as the modulus of the flux vector at each position (black indicating fluxes close to zero, while solid colour indicates maximum flux values within the field). **(J)** Basic model; **(K)** import-dependent model; **(L)** the same model as (K), but with import augmented 20-fold in relation to default import (P_{IAAH}=5 µm/s; P_{LAX1}=100 µm/s); **(M)** export-dependent model; **(N)** combined model, implementing changes of both (K) and (M). Note that only the scenarios (L–N) give rise to an auxin minimum, with the distinction that in (L) there are no fluxes through the VM (indicated by the dark colour). Hence, the terminology "flux-barrier" mechanism displayed by the import-based model (L) and "flux-passage" mechanism by the export-based model (M). **(O–S)** Magnification of a portion of the fruit tissue (with the VM), to better visualise the fluxes through the VM, where **(O)** shows (J), **(P)** shows (K), **(Q)** shows (L), **(R)** shows (M), and **(N)** shows (S). Colour coding of auxin levels as indicated in Figure 2.

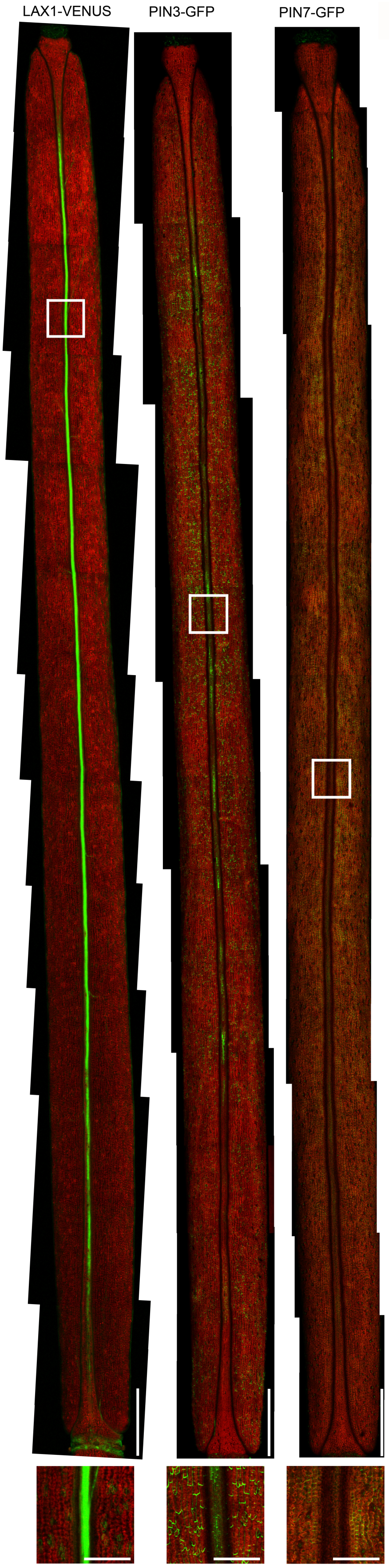

Figure S4: **Supporting data on transporter localisations at stage 17b. Related to Table 2.** **(A)** *LAX1::LAX1-VENUS*; **(B)** *PIN3::PIN3-GFP*; **(C)** *PIN7::PIN7-GFP*, with zoomed-in regions below. Scale bars: 1 mm for (A–C); 200 μm for lower panels.

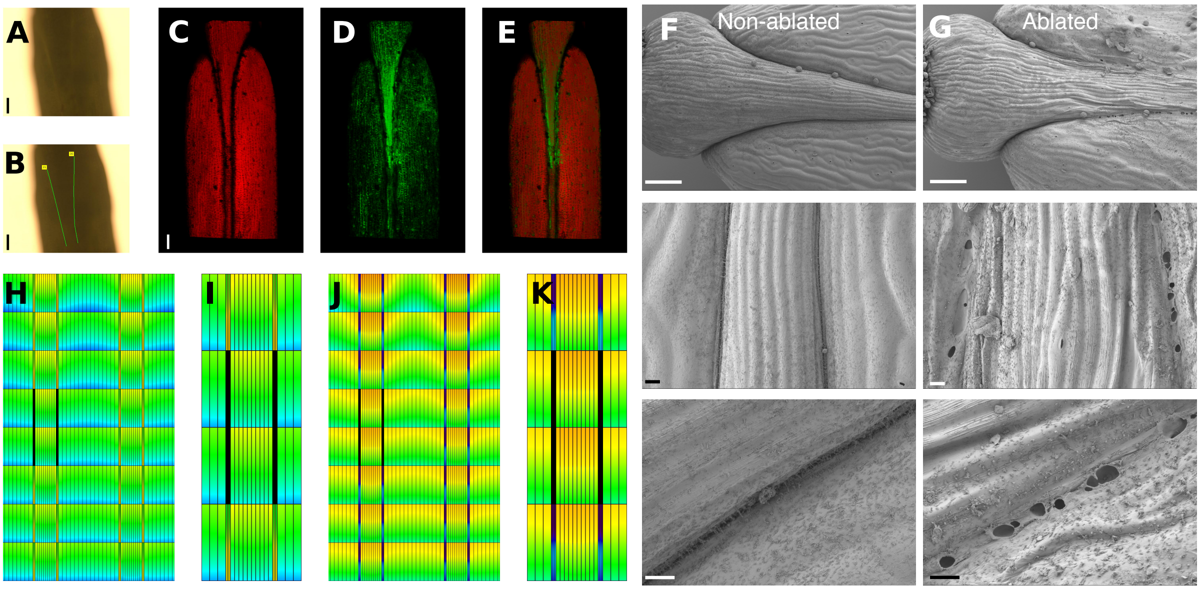

Figure S5: **Laser ablation and partial *in silico* ablation. Related to Figure 4. (A, B)** Whole-mount image of apical part of stage-15 fruit (A) with sites of ablation indicated (B). **(C–E)** Confocal scans 7 days after ablation show autofluorescence (C), DR5::GFP (D) and merge (E). Scanning electron micrographs of tissue before **(F)** and after **(G)** laser ablation. Note the holes in cells of panel (G) caused by the laser. Scale bars: top row 100 μm; middle and bottom row 10 μm. **(H)** Flux-passage mechanism reacting to a small ablation; **(I)** flux-barrier mechanism reacting to a small ablation; **(J)** zoomed in portion of (H); **(K)** zoomed in portion of (I); Scale bars: 100 μm. Colour coding of auxin levels as indicated in Figure 2.

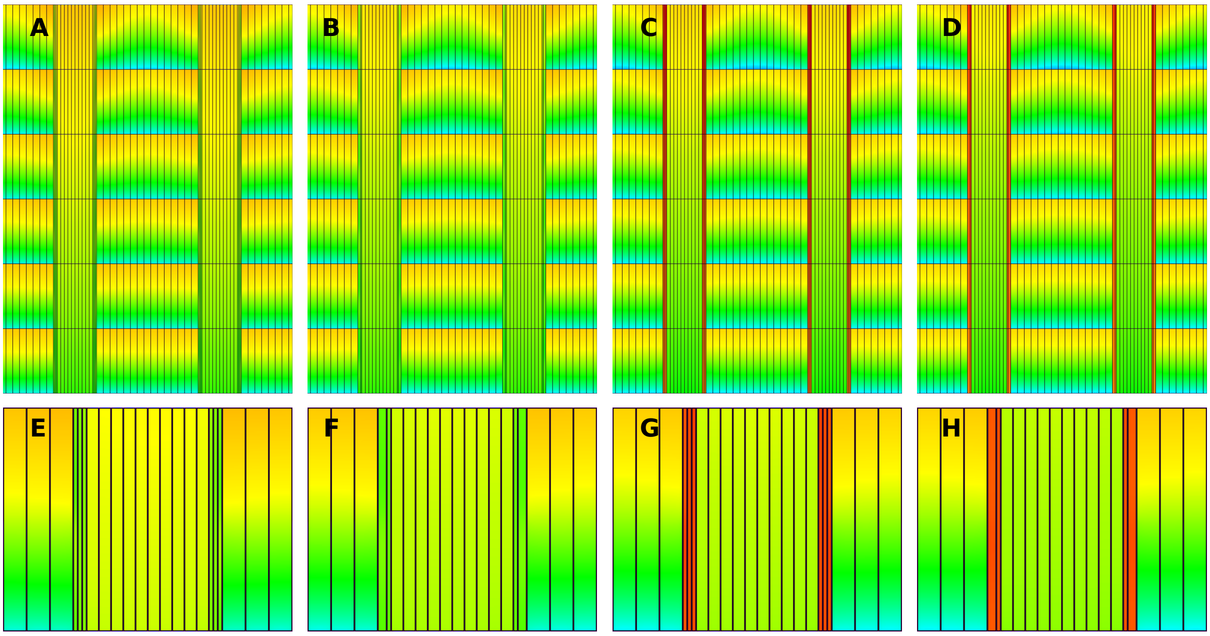

Figure S6: **Auxin patterning progression from stage 15 to 16. Related to Figure 5. (A)** stage 16; **(B)** tissue layout of stage 15 (i.e., one less VM cell file), but PIN expression as in stage 16; **(C)** stage 15, considering only the PIN differences, as given in Table 2; **(D)** stage 15, taking both PIN (C) and tissue (B) alterations into account. Zoomed in views **(E)** of pattern (A); **(F)** of pattern (B)**; (G)** of pattern (C)**; (H)** of pattern (D). Colour coding of auxin levels as indicated in Figure 2.

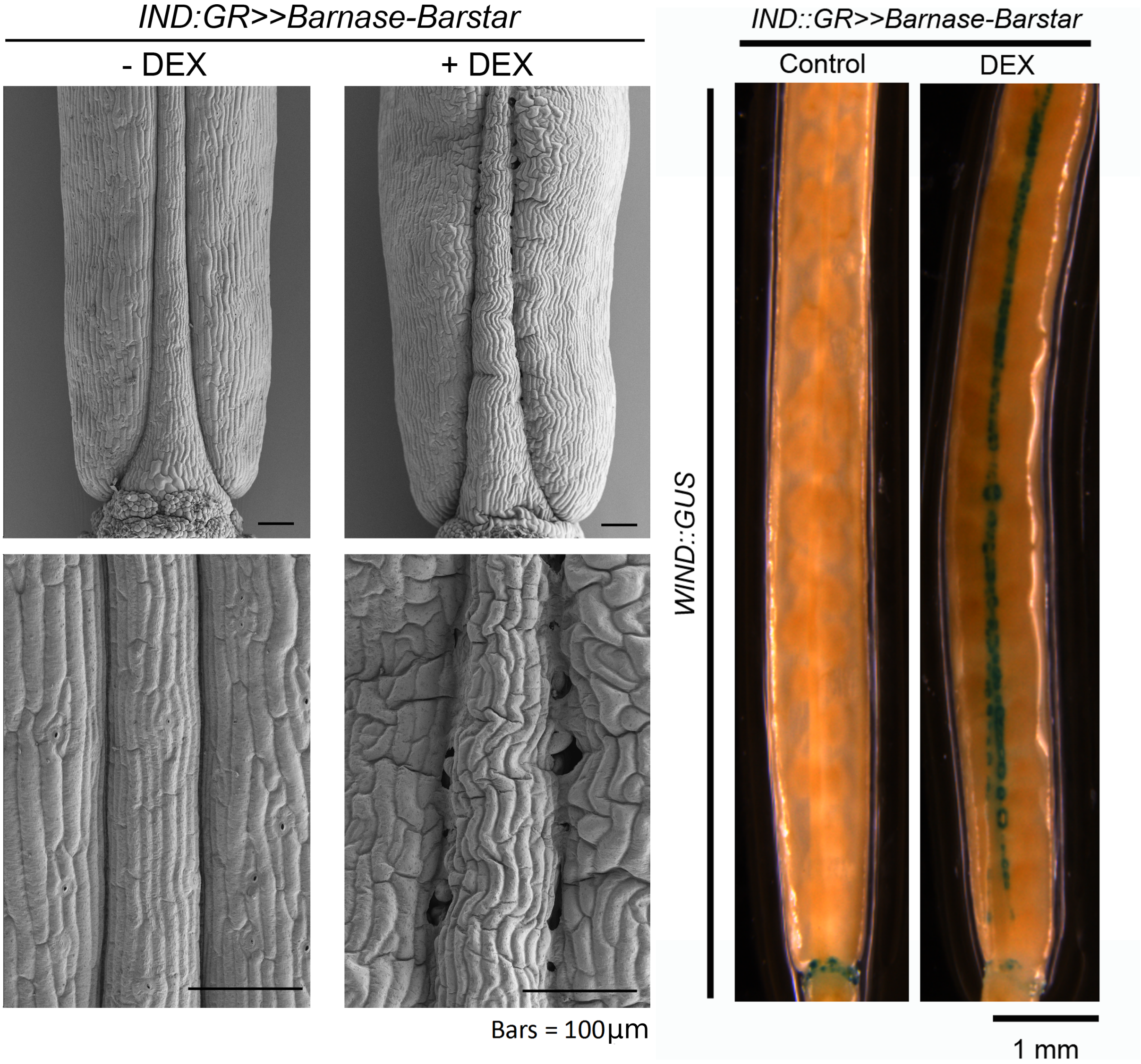

Figure S7: **VM removal with localised wound response. Related to Figure 4.** (left panels) Damage to tissue due to the induced, localised cell death of the VM cells. Scale bars: 100 μm. (right panels) The localised VM wound response is indicated through WIND1::GUS expression after induction. Scale bar 1 mm.

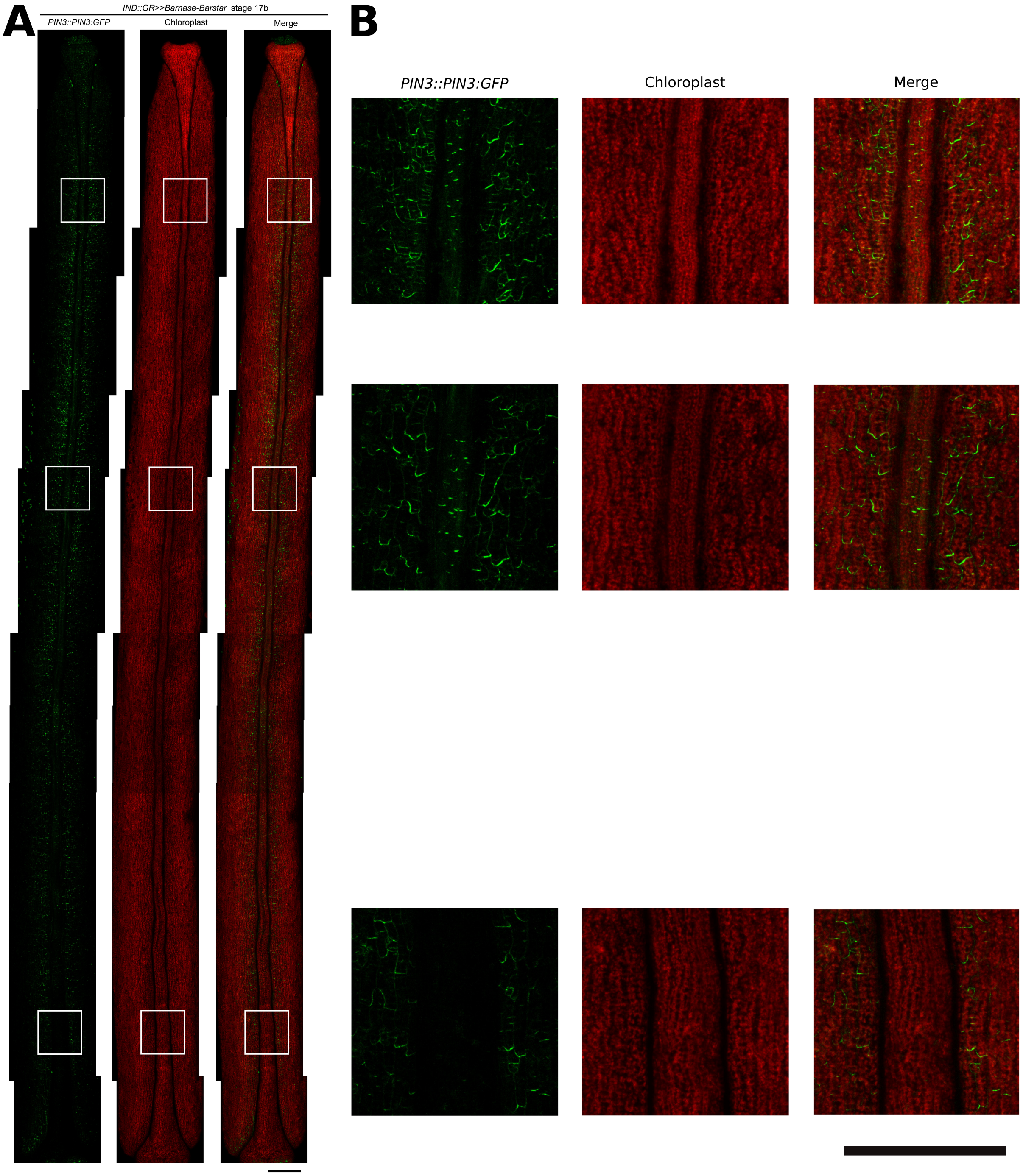

Figure S8: **PIN3 expression patterns are not altered following chemical ablation. Related to Figure 4. (A)** PIN3::PIN3-GFP under the IND>GR>BARNASE-BARSTAR system, after 9 days of treatment (left), chloroplast (middle), and merged (right). **(B)** Detailed insets from (A), as indicated. Both scale bars are 400 μm.

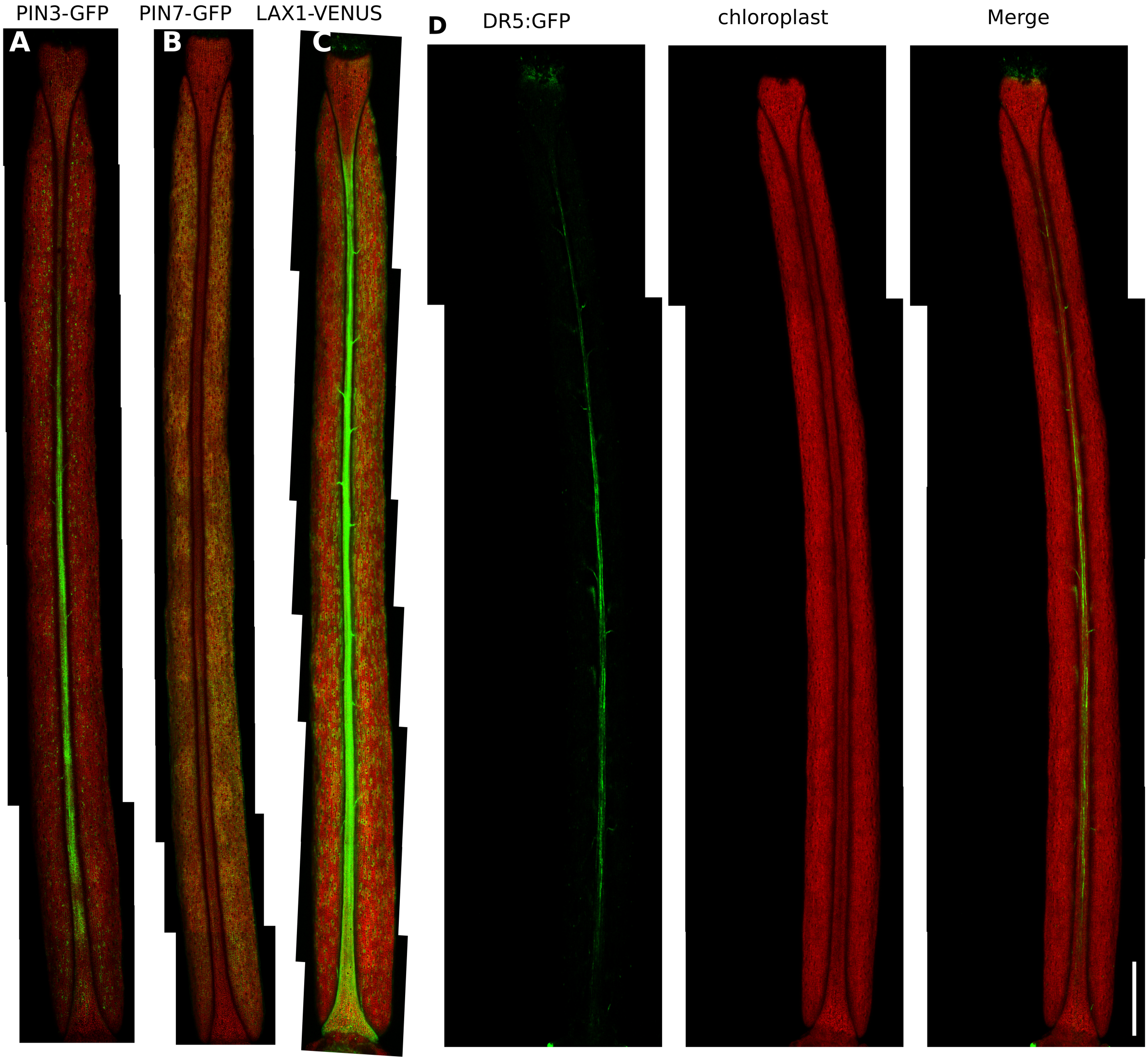

Figure S9: **Expression patterns at stage 16. Related to Table 2.** (**A**) PIN3::PIN3-GFP; (**B**) PIN7::PIN7-GFP; (**C**) LAX1::LAX1-VENUS; and (**D**) DR5::GFP (left), chloroplast (middle), and merged (right). Scale bar 1 mm.

	Parameter	Unit	Description	Replum	Separation layer	Lignifying layer	Valve
simplified tissue							
	l_c	µm	Cell width	10	3	3	20
	h_c	µm	Cell height	200	200	200	200
	n_l	—	Cell files	10	1	2	15
	n_h	—	Cell rows	12	12	12	12
realistic tissue							
	l_c	µm	Cell width	10	3	3	20
	h_c	µm	Cell height	200	200	200	200
	n_l	—	Cell files	10	1	2	65
	n_h	—	Cell rows	75	75	75	75

Table S1: **Parameter values related to tissue layout.** The conceptual models were simulated using the simplified tissue layout, while the detailed, realistic simulations were done using the realistic tissue layout scaled to our own measurements using microscopy images at stage 17b.

	Parameter	Unit	Description	Value
Auxin availability and diffusion				
	a_0	[a.u.]/μm^2	Auxin concentration at the apical boundary	0.1
	D	μm^2/s	Auxin diffusion constant	600
Auxin transport				
	P_{IAAH}	μm/s	Passive influx permeability	20
	M_{LAX1}	μm/s	Maximum LAX1 permeability	20
	P_{IAA^-}	μm/s	Passive efflux permeability	1
	M_{PIN3}	μm/s	Maximum PIN3 permeability	10
	M_{PIN7}	μm/s	Maximum PIN7 permeability	10
Auxin biosynthesis				
	δ_{IAA}	s^{-1}	Auxin decay rate	$5 \cdot 10^{-7}$
	b	[a.u.]/(μm^2·s)	Auxin biosynthesis rate	$5 \cdot 10^{-5}$
Cell wall				
	w_l	μm	Cell wall width	1
Numerical details				
	Δx	μm	Numerical space step	1
	Δt	s	Numerical time step	0.1

Table S2: **Default parameter values.** The values used in the simulations except when indicated otherwise.

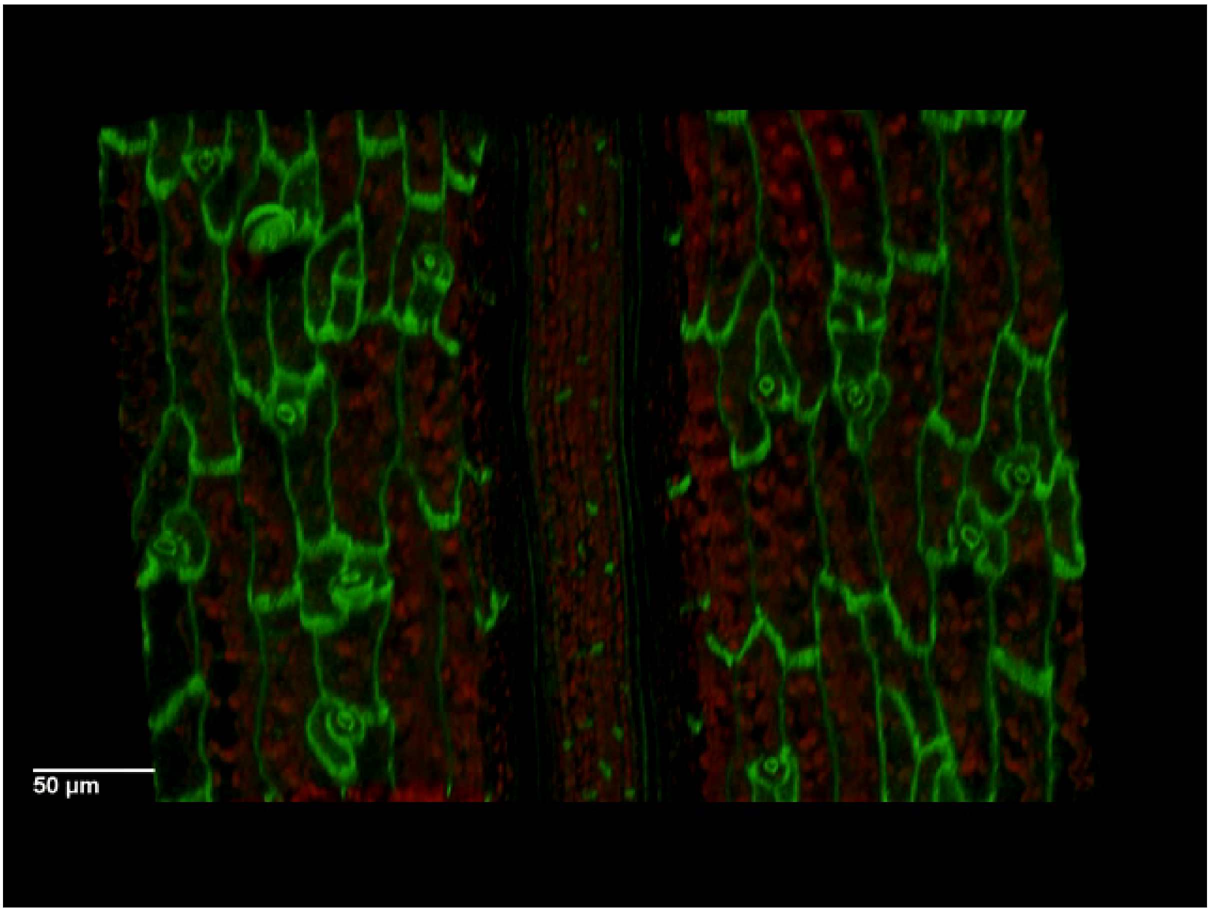

Movie S1: **PIN3 distributions in the VM.** 3D imaging reveals PIN3 apolar localisation to the cells of the VM.
